# Biodiversity of coral reef cryptobiota shuffles but does not decline under the combined stressors of ocean warming and acidification

**DOI:** 10.1073/pnas.2103275118

**Published:** 2021-09-20

**Authors:** Molly A. Timmers, Christopher P. Jury, Jan Vicente, Keisha D. Bahr, Maryann K. Webb, Robert J. Toonen

**Affiliations:** ^a^Pristine Seas, National Geographic Society, Washington, DC 20036;; ^b^Hawai‘i Institute of Marine Biology, School of Ocean and Earth Science and Technology, University of Hawai‘i at Mānoa, Honolulu, HI 96744;; ^c^Department of Life Sciences, Texas A&M University–Corpus Christi, Corpus Christi, TX 78412

**Keywords:** cryptobenthic, ARMS, COI metabarcoding, climate change, mesocosm

## Abstract

Ocean-warming and acidification are predicted to reduce coral reef biodiversity, but the combined effects of these stressors on overall biodiversity are largely unmeasured. Here, we examined the individual and combined effects of elevated temperature (+2 °C) and reduced pH (−0.2 units) on the biodiversity of coral reef communities that developed on standardized sampling units over a 2-y mesocosm experiment. Biodiversity and species composition were measured using amplicon sequencing libraries targeting the cytochrome oxidase I (COI) barcoding gene. Ocean-warming significantly increased species richness relative to present-day control conditions, whereas acidification significantly reduced richness. Contrary to expectations, species richness in the combined future ocean treatment with both warming and acidification was not significantly different from the present-day control treatment. Rather than the predicted collapse of biodiversity under the dual stressors, we find significant changes in the relative abundance but not in the occurrence of species, resulting in a shuffling of coral reef community structure among the highly species-rich cryptobenthic community. The ultimate outcome of altered community structure for coral reef ecosystems will depend on species-specific ecological functions and community interactions. Given that most species on coral reefs are members of the understudied cryptobenthos, holistic research on reef communities is needed to accurately predict diversity–function relationships and ecosystem responses to future climate conditions.

## Significance

Although climate change is expected to decimate coral reefs, the combined impacts of ocean-warming and acidification on coral reef biodiversity remains largely unmeasured. Here, we present a two-year mesocosm experiment to simulate future ocean acidification and ocean-warming to quantify the impacts on species richness, community composition, and community structure. We find that species richness is equivalent between the dual-stressor and present-day treatments but that the community shuffles, undoubtedly altering ecosystem function. However, our ability to predict the outcomes of such community shuffling remains limited due to the critical knowledge gap regarding ecological functions, life histories, and distributions for most members of the cryptobenthic community that account for the majority of the biodiversity within these iconic ecosystems.

As the concentration of atmospheric carbon dioxide (pCO_2_) continues to rise, marine biodiversity is predicted to decline due to ocean-warming and acidification (1). Warming seas and increased acidity are expected to disproportionately affect marine ecosystems built by calcifying biota (2–4). Coral reefs are among the most sensitive marine ecosystems affected by global stressors, because the primary ecosystem engineers, calcifying scleractinian corals and coralline algae, show direct physiological responses to both elevated temperature and acidification, resulting in strong indirect effects on habitat structure and community composition (5, 6). In this century alone, record-breaking sea surface temperature anomalies have resulted in widespread coral mortality (7, 8), leading to a reduction in topographic complexity (9) and a shift in community composition (10, 11). Likewise, in situ observations of coral reefs along naturally occurring gradients of acidification have shown declines in habitat complexity (5, 6) and diversity (12, 13), as well as changes in community structure (14, 15). The combination of both thermal stress and acidification stress over the coming decades is predicted to have synergistic negative effects on reef resilience (2, 3, 16) by eroding the reef framework (17), shifting the structural dominance away from calcifiers and severely diminishing the biodiversity of this iconic ecosystem (2, 4). Coral reefs occupy less than 1% of the seafloor but house over 25% of all marine species; the loss of biodiversity due to anthropogenic stressors is predicted to lead to the functional collapse of these ecosystems later this century (2, 4, 18, 19). However, future projections of the combined effects of increased temperature and acidity on biodiversity have typically been derived from reviews and meta-analyses based on short-term, single-species experimental manipulations (20–23) or from in situ observations of a handful of taxa along natural gradients of seawater chemistry or temperature (5, 7, 12, 13, 24).

Although such studies have informed our understanding of how some reef communities may change in the future, tradeoffs also exist for each approach in understanding climate impacts on biodiversity. Natural gradient studies do not simultaneously incorporate end-of-the-century levels of both acidification and warming, and short-term perturbation experiments are typically performed over days to weeks on single focal species. While short-term perturbation experiments across life stages have been instrumental in understanding how changes in ocean temperature, chemistry, and their combined effects influence organismal physiology (25, 26), they do not include diurnal or seasonal environmental changes (27–29) or realistic multispecies communities, which inherently excludes the roles of environmental variation and ecological interactions from contributing to the measured responses. Species interactions could be critical to experimental outcomes because they can modify population growth rates, behavior, consumption, reproduction, production, the efficacy of defensive structures, and resource availability, thereby influencing species densities, composition, and richness through competition, facilitation, or predation (30–34). Ultimately, species interactions determine whether ecosystem functions are maintained or diminished under altered environmental conditions (35–37). Thus, there is a pressing need for long-term, multispecies experimental work to understand the responses of complex communities to future climate change scenarios.

Here, we examined the independent and combined effects of ocean-warming and acidification on the biodiversity of coral reef communities in long-term (2-y) mesocosms. In experimental flow-through mesocosms that received unfiltered seawater drawn from an adjacent reef slope, we examined the cryptobiota communities that developed on standardized habitats (two-tiered Autonomous Reef Monitoring Structures, or ARMS) (38) in each of four treatments: present-day pH and temperature (Control treatment), ocean acidification (−0.2 pH units—Acidified treatment), ocean-warming (+2 °C—Heated treatment), and future ocean combined stressors (−0.2 pH units and +2 °C—Acidified-Heated treatment) (*SI Appendix*, Fig. S1). These experimental ocean-warming and acidification conditions reflect those predicted for the late 21st century given current commitments under the Paris Climate Accord (roughly intermediate between Representative Concentration Pathways RCP 6.0 and RCP 8.5) (39).

Each mesocosm was initially established with a 2-cm layer of carbonate reef sand and gravel as well as pieces of reef rubble (three replicate 10- to 20-cm pieces randomly divided among mesocosms) collected from the adjacent reef, thereby including natural infaunal and surface-attached communities. A juvenile (3- to 8-cm) Convict surgeonfish (*Acanthurus triostegus*), a generalist grazer on benthic algae, a Threadfin butterflyfish (*Chaetodon auriga*), a generalist grazer on noncoral invertebrates, and five herbivorous reef snails (*Trochus* sp.) were added to each tank to provide the essential ecological functions of herbivory and predation in the mesocosms at biomass values approximating Hawaiian reefs (40). Finally, the eight regionally most common reef-building coral species (*Montipora capitata*, *Montipora flabellata*, *Montipora patula*, *Pocillopora acuta*, *Pocillopora meandrina*, *Porites compressa*, *Porites evermanni*, and *Porites lobata*) were added as small fragments to each the mesocosms for an initial coral cover of ∼10% to begin the experiment. The corals and rubble were placed on a plastic grate 6 cm above the sediments to simulate their attachment to hard substrate in nature, and the ARMS were placed underneath the grate to simulate the location of the cryptobenthic habitat (*SI Appendix*, Fig. S2). Among the added species, only one species of coral was extirpated from a single treatment. Thus, we target the cryptobenthic community here, because they comprise the vast majority of biodiversity on coral reefs (41) and show significant community responses to our experimental treatments. Furthermore, due to the challenges associated with surveying the cryptobiota using visual census, these organisms are often overlooked in coral reef climate change research despite their essential roles in nutrient cycling, cementation, trophodynamics, and other ecological processes (42–45). As studies are increasingly pointing toward the critical functional importance of this community in food webs and the maintenance of biodiversity on coral reefs (43, 45, 46), there is a need to diminish the existing knowledge gap on both taxonomic composition and ecosystem function of this community in response to climate change.

After two years of exposure, we examined the coral reef community that had developed on each ARMS unit. We generated amplicon sequence libraries targeting cytochrome oxidase I (COI) (the most extensive barcode database currently available) from each unit to test whether species richness, community composition (occurrence), or community structure (relative abundance) of the cryptobenthic community changed with treatment. This experimental study evaluates the richness and composition of an entire coral reef community which developed over a multiyear time frame under predicted future ocean conditions.

## Results

**Fig. 1.**
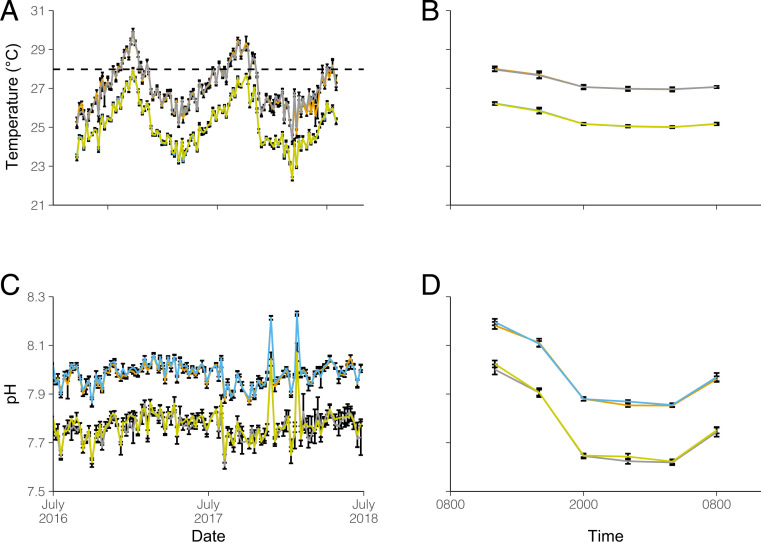
Environmental data from the mesocosm experiment. Time series of the daily mean temperature (*A*) and pH (*C*) and the hourly mean temperature (*B*) and pH (*D*) over the diel cycle (mean ± SD). Data are based on weekly sampling at 1,200 h as well as monthly sampling every 4 h over the diel cycle (*SI Appendix*). The horizontal dashed line (*A*) shows the nominal coral bleaching threshold. Treatments are colored as follows: Control—blue, Acidified—yellow, Heated—orange, and Acidified-Heated—gray.

**Fig. 2.**
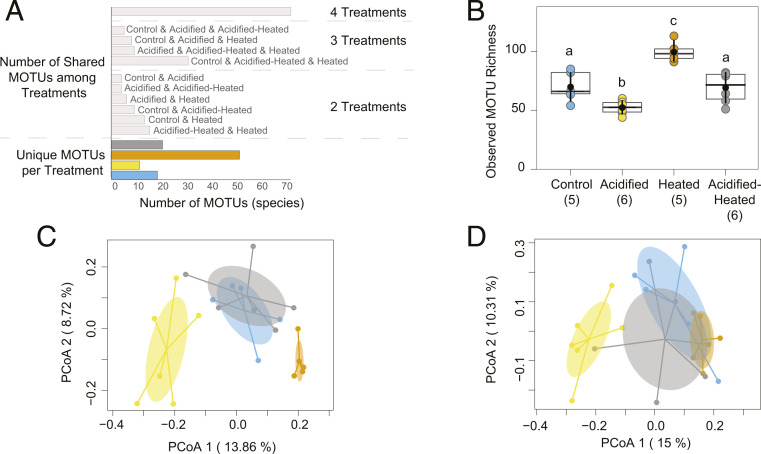
Species richness represented by shared, unique, and overall MOTUs per treatment and treatment communities visualized through principal coordinate analysis (PCoA). (*A*) MOTU distribution (*n* = 275) based on the number of shared MOTUs among treatments as well as the number of unique MOTUs per treatment. (*B*) Observed MOTU richness by treatment. Black dots represent mean richness, the crosshatch is the median, box limits are upper and lower quartiles, and the vertical lines through the mean represent one SD above and below the mean. Parentheses represent the number of ARMS units within each treatment. Lowercase letters denote significant differences among treatments at alpha = 0.05 based on Tukey post hoc results. (*C*) PCoA on community composition (present/absent—Jaccard dissimilarity index) and (*D*) PCoA on community structure (relative abundance—Bray–Curtis dissimilarity index). Ellipses are colored by treatment and based on the 95% confidence limit of the SEM for each group. Colored dots represent ARMS units within treatments. Treatments are colored as follows: Control, blue; Acidified, yellow; Heated, orange; and Acidified-Heated, gray.

Temperature and pH in all mesocosms followed natural diel and seasonal variations similar to those experienced on the reef (Table 1 and Fig. 1), and over the 2-y experimental cycle, mesocosms with elevated temperature (Heated and Acidified-Heated) experienced temperatures at or above the nominal bleaching threshold for 3.5 mo each year with an annual accumulation of 24 Degree Heating Weeks (DHW). Under these conditions, a total of 275 species proxies, representative molecular operational taxonomic units (MOTUs), from 354,921 rarefied sequences, representing 13 phyla were obtained from 22 ARMS units after the 24-mo soaking period within the mesocosms (*SI Appendix*, Tables S1–S3). Taxonomic composition consisted predominantly of seven phyla across treatments, representing 95% of the MOTUs and 99.7% of the sequences (Annelida, Arthropoda, Cnidaria, Echinodermata, Mollusca, Porifera, and Rhodophyta). Only 26 MOTUs (∼10%) could be identified to a species level (*SI Appendix*, Table S4), highlighting the extremely limited number of vouchered marine barcode references currently available from this diverse coral reef community (*SI Appendix*, Fig. S3). There were 54 families identified from 134 MOTUs; however, over 50% of the MOTUs could not be identified to the family level. In total, 25% of the MOTUs (*n* = 70) were found within all treatments, and 36% (*n* = 99) were unique to a single treatment with roughly 50% of unique MOTUs being found in the Heated treatment (Fig. 2*A*). The remaining 39% (*n* = 110) were shared among at least two treatments with the Acidified treatment sharing the least among all treatments.

**Table 1.** Carbonate chemistry and temperature from the experiment

Treatment	Salinity (psu)	Temperature (°C)	pH	Total alkalinity (µmol/kg^−1^)	pCO_2_ (µatm)	Ω_arag_
Control	34.24 ± 0.35 (0.01)	25.10 ± 1.24 (0.09)	7.99 ± 0.06 (0.01)	2,176 ± 54 (11)	447 ± 61 (13)	2.88 ± 0.34 (0.05)
Acidified	34.24 ± 0.35 (0.02)	25.12 ± 1.23 (0.09)	7.78 ± 0.07 (0.02)	2,184 ± 51 (12)	798 ± 128 (41)	1.93 ± 0.30 (0.08)
Heated	34.27 ± 0.35 (0.02)	27.05 ± 1.20 (0.16)	7.98 ± 0.06 (0.01)	2,188 ± 51 (11)	455 ± 62 (15)	3.06 ± 0.35 (0.07)
Acidified-heated	34.27 ± 0.35 (0.02)	27.11 ± 1.50 (0.15)	7.77 ± 0.07 (0.02)	2,197 ± 50 (9)	819 ± 129 (46)	2.06 ± 0.31 (0.08)

Data are daily mean values based on weekly sampling at 12:00 h as well as monthly sampling every 4 h over the diel cycle (*SI Appendix*) and are shown as mean ± SD. The uncertainties associated with these values reflect variation from day to day, seasonally, as well as among replicate mesocosms in each treatment. The mean uncertainty among mesocosms on a given sampling day is provided in parentheses. Note that the variation among mesocosms is relatively small, and most of the variation is explained by daily and seasonal fluctuation of these parameters.

Elevated temperature had a positive effect (ANOVA: *f*_1,14_ = 28.33, *P* < 0.001), pH had a negative effect (ANOVA: *f*_1,14_ = 29.77, *P* < 0.001), and the combined stressors had no effect on MOTU richness (ANOVA: *f*_1,14_ = 2.36, *P* = 0.146) (Fig. 2*B* and *SI Appendix*, Tables S5 and S6). There was no difference in richness between the Control and Acidified-Heated treatments (Tukey post hoc: *t*_14_ *=* 0.14, *P* = 0.99) (Fig. 2*B* and *SI Appendix*, Table S7), and there was no effect of header tanks (ANOVA: *f*_4,14_ = 1.19, *P* = 0.355).

Community composition, defined as MOTU present/absent, varied under elevated temperature ( permutational multivariate analysis of variance [PERMANOVA] *pseudo f*_1,18_ = 1.89; *R*^2^ = 0.08; *P* = 0.001)), pH (*pseudof*_1,18_ = 1.94; *R*^2^ = 0.08, *P* < 0.001) and the interaction of elevated temperature and pH (*pseudof*_1,18_ = 1.53; *R*^2^ = 0.06, *P* = 0.02) (Fig. 2*C* and *SI Appendix*, Fig. S4). However, there was no difference in community composition between the Control and Acidified-Heated treatments (pairwise PERMANOVA: *P* = 0.62) (*SI Appendix*, Table S8). The variation in community composition was not attributed to differences among within-group variability [permutational analysis of multivariate dispersion (PERMDISP): *P* > 0.05, *SI Appendix*, Table S9 and Fig. S5*A*]; however, the Heated treatment was more variable than the other treatments (pairwise PERMDISP: *P* = < 0.05) (*SI Appendix*, Table S10).

Community structure, defined by the relative abundance of sequences for each MOTU, varied with elevated temperature (PERMANOVA: *pseudof*_1,18_ = 2.06; *R*^2^ = 0.09; *P* = 0.002), pH (*pseudof*_1,18_ = 2.22; *R*^2^ = 0.09, *P* < 0.001) and the interaction of elevated temperature and pH (*pseudof*_1,18_ = 1.54; *R*^2^ = 0.06, *P* = 0.03) (Fig. 2*D*). For community structure, pairwise comparisons showed significant differences among all treatments (*SI Appendix*, Table S8). Within-group variability was not different among treatments (PERMDISP: *P* > 0.72) (*SI Appendix*, Table S9 and Fig. S5*B*).

**Fig. 3.**
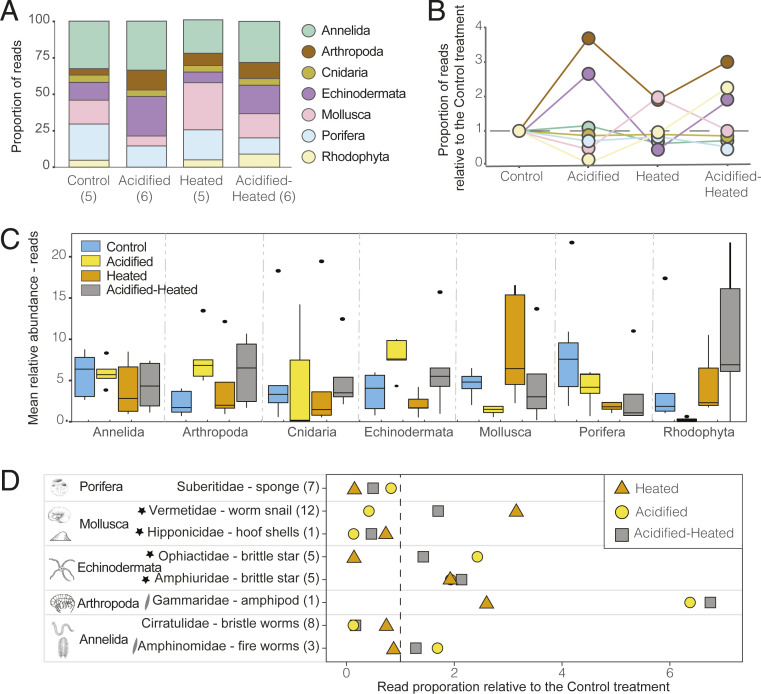
Variation in the top seven most abundant phyla and the top eight most abundant families among treatments. (*A*) Proportion of reads by phyla within treatments with the number of sampling units in parentheses. (*B*) Proportion of reads by phyla among treatments relative to the present-day (Control) condition. (*C*) Relative abundance of phyla among treatments. Box plots show the median as the center line, box limits are upper and lower quantiles, whiskers are 1.5× interquartile range, and open circles represent outliers. (*D*) The eight identified families that each represent >4% of total reads, showing the proportion of reads compared to present-day (Control—represented by the vertical line at x = 1). Parentheses next to families represents the number of MOTUs within that family, the stars represent heavily calcifying families, and the slanted lines symbol represents families with limited calcification. The number of reads and MOTUs from these eight families represent 53% and 15% of the total, respectively.

Different taxonomic groups dominated the cryptobenthic community within each treatment (Fig. 3 and *SI Appendix*, Tables S11 and S13; refer to *SI Appendix*, Fig. S6 and Table S12 for MOTUs). Acidification had a positive effect on the relative abundance of arthropods (permutational ANOVA: *f*_1,14_ = 4.34, *P* = 0.05) and echinoderms (permutational ANOVA: *f*_1,14_ = 8.22, *P* = 0.02). Relative to the Control condition, they were two to three times more abundant under Acidified conditions (Fig. 3*B* and *SI Appendix*, Table S11; refer to *SI Appendix*, Fig. S7 for an echinoderm example). Elevated temperature conditions had a positive effect on the red algae (rhodophytes) (permutational ANOVA: *f*_1,14_ = 3.34, *P* = 0.08), while acidification had no effect (permutational ANOVA: *f*_1,14_ = 0.02, *P* = 0.89). However, this lack of significance under reduced pH is most likely due to an interaction between temperature and pH (permutational ANOVA: *f*_1,14_ = 3.89, *P* = 0.06) (*SI Appendix*, Table S13). Compared to the Control, rhodophyte read abundance more than doubled in the Acidified-Heated treatment, whereas these algae were nearly missing within the Acidified treatment (0.04%) (Fig. 3*C* and *SI Appendix*, Fig. S8 and Table S11).

Of the 53 families identified, eight had >4% of the total reads, and together, these eight families accounted for 53% of the reads and 15% of the MOTUs. Of these eight families, four heavily calcify (Vermetidae and Hipponicidae—phylum Mollusca; Ophiactidae and Ampiuridae—phylum Echinodermata), two have limited calcification (Gammaridae—phylum Arthropoda; Amphinomidae—phylum Annelida), and two do not calcify (Suberitidae—phylum Porifera; Cirratulidae—phylum Annelida). Worm snails (Vermetidae) showed increased read abundance in the Heated and Acidified-Heated treatments relative to the Control, with elevated temperature having a positive effect on their prevalence (permutational ANOVA: *f*_1,14_ = 4.88, *P* = 0.05) (*SI Appendix*, Fig. S9), whereas hoof shells (Hipponicidae) were less likely to persist under acidification (permutational ANOVA: *f*_1,14_ = 6.12, *P* = 0.02) (Fig. 3*D* and *SI Appendix*, Table S14). Acidification had a positive effect on the brittle stars, Ophiactidae (permutational ANOVA: *f*_1,14_ = 5.11, *P* = 0.04) and Amphiuridae (permutational ANOVA: *f*_1,14_ = 3.10, *P* = 0.09, respectively) with abundances 1.5 to 2.5 times greater under acidification as compared to present-day conditions. Gammarid amphipods prevailed under acidification (permutational ANOVA: *f*_1,14_ = 11.43, *P* = < 0.01) with more than six times the numbers of reads in the Acidified treatments as compared to the Control. The noncalcifying sponges from Suberitidae did not favor elevated temperature (permutational ANOVA: *f*_1,14_ = 3.75, *P* = 0.06) (*SI Appendix*, Fig. S10), whereas the noncalcifying bristle worms (Cirratulidae) were inhibited by acidification (permutational ANOVA: *f*_1,14_ = 4.79, *P* = 0.02).

## Discussion

Predictions about massive loss of coral reef biodiversity by the end of the century have been largely extrapolated from single-species response experiments or from observations based on patterns of surface-dwelling macrofauna along localized natural gradients of either temperature or pH (5, 7, 12, 13, 20–22). Our results suggest that such experiments and observations may not scale directly to the response of a complex community. Predicting the future of coral reef biodiversity and ecosystem function is more complicated than summarizing the net effects of warming and acidification on a handful of species studied in isolation. Reefs of the future will undoubtedly differ from those of today, but in terms of overall biodiversity, a drastic decline in species richness is inconsistent with results from our experimental mesocosms. Our analyses indicate that increased temperature and increased acidification have opposing effects on species richness, such that the communities which develop under the combination of warming and acidification expected by the end of the century have equivalent richness as compared to present-day conditions. This result goes against the current paradigm that the interaction of warming and acidification should have either additive or synergistic negative effects on reef biodiversity and function (2, 47, 48). However, much of the evidence in support of the current consensus stems from experiments and observations that have focused predominantly on calcifying organisms that are of most concern, such as corals and coralline algae, rather than a representative subset of the diverse species pool which inhabits coral reefs. Further, most of these experiments do not consider species interactions or system responses that follow natural biological rhythms and take extended periods of time to develop.

Contrary to predictions, there is evidence from both individual and multispecies experiments that increases in temperature and acidity can have counteracting effects on organismal physiology. For example, low levels of warming have been found to ameliorate the negative effects of acidification on the calcification, growth, and recruitment rates of some organisms (49–51), and acidification has been found to mitigate the consequences of elevated temperature on recruitment and body size in others (52, 53). Physiological buffering (54, 55) and trophic compensation among species have also been reported to mediate the effects of warming and acidification on community composition (31, 56). We did not examine treatment effects on individual species in this study, but given that the community composition was most similar between the Control and future ocean dual-stressor treatments, some such amelioration and/or compensation under the combined factors is also likely in effect here.

Unlike the future ocean dual-stressor treatment, the individual effects of ocean-warming and acidification had stronger influences on species richness, with richness significantly reduced under acidification but elevated under ocean-warming despite the annual accumulation of 24 DHW. For many marine invertebrate organisms, warming increases metabolism across life stages with faster growth when temperatures are up to 4 °C above ambient thermal history (26). The enhanced richness within the +2 °C Heated treatment could be a result of increasing rates of growth and development of cryptobiota under warming, thereby increasing the probability of successful settlement or survival. Acidification, on the other hand, has been found to have more direct negative effects across a wider range of phyla and life stages than elevated temperature (21–23) with calcifying larvae being particularly vulnerable to lethal and sublethal responses during development (23, 26, 57). Acidification-induced changes in larval development times, dispersal distances, and shifts in microbial biofilms used as settlement cues for larvae have also been shown to alter recruitment and settlement dynamics (58–61), all of which could have contributed to the diminished richness observed within the Acidified treatment.

Even though these communities were all derived from the same species pool, we find that community composition and community structure differed across treatments, with the exception of similar species composition under both the end-of-century and present-day ocean conditions. Communities that developed in the Heated and the Acidified treatments did not overlap with each other or with the other treatments, suggesting strong differential responses in larval development, metamorphosis, survivorship, reproductive strategies, or competitive interactions under these conditions. The competitive landscape appears to shift among treatments such that different taxonomic groups come to dominate the communities within each treatment. A similar result was obtained from an experimental warming study in Antarctica, which showed that over the course of 9 mo, a 1 to 2 °C rise in temperature favored divergent taxa and resulted in distinctive species assemblages on warmed settlement panels relative to the control (62). Shifts in competitive dominance have also been found on settlement plate assemblages across natural gradients in acidification such that calcifiers were consistently replaced by fleshy algae under increasing acidity (15, 63, 64). In contrast, this study found differing responses among diverse calcifying taxa under reduced pH.

For the heavily calcifying phyla Mollusca and Echinodermata, mollusks were often losers under acidification, while echinoderms (ophiactid and amphiurid brittle stars) were consistently winners.

The high relative abundance of brittle stars found within the Acidified treatments could be a result of the reproductive strategies found within these families. Ophiactids can reproduce both sexually (broadcast spawning) and asexually (fissiparity), and members within this family have been found to initiate asexual reproduction when stressed from external stimuli (65, 66). Some amphiurid species, such as those within the *Amphipolis squamata* complex identified in these mesocosms (*SI Appendix*, Table S4), are known brooders. Taxa that brood or have direct development appear to have an advantage to survival and reproduction in acidified waters, because juveniles are minimally exposed to the environmental conditions (67). While maternal care may drive the dominance of brittle stars under Acidified conditions, this strategy does not appear to be advantageous for all brooding taxa. Among the mollusk families, some hipponicids (hoof shells) and all vermetids (worm snails) provide maternal care. However, hipponicids universally struggled under Acidified conditions, whereas vermetids (worm snails) were reduced in the Acidified treatment but thrived in both the Acidified-Heated and Heated treatment, suggesting that a warming compensatory mechanism was at play for this group. While nonspawning/nonlarval life cycles may allow some species to opportunistically occupy space quickly, clearly, organismal responses to acidification and warming are not easily generalized based on overall taxonomic characterization, degree of mineralization, life history strategy, or functional group but rather appear to be driven by species-specific tolerances which vary within each phylum.

Competitive release may also influence organismal responses to acidification. Gammarid amphipods flourished under acidification with proportional biomass over three times greater than in the Control treatment. These micrograzers exhibited similar patterns along an acidified rocky reef vent system, in which the greatest densities of amphipods were found at the low pH sites (68). As amphipods are direct developers, maternal care could be a factor resulting in their abundance in acidified environments. However, like our Acidified treatment, the acidified rocky vent sites had lower species richness relative to ambient, and it was suggested that either competitive release or a decrease in predation rates were driving higher amphipod abundance. These mechanisms may also help to explain the dominance of amphipods under low pH within our experiment.

Other groups, such as sponges and red algae (rhodophytes), showed unexpected sensitivity to future ocean conditions but not predictably. Noncalcifying sponges had half the read abundance in the dual-stressor treatment relative to the Control. Rhodophytes had the greatest read abundance in the Acidified-Heated treatment but were rare in the Acidified treatment. Because both fleshy and calcified rhodophytes colonized these mesocosms, it is surprising that even fleshy species were largely absent from the Acidified treatment. These results are consistent with the variety of studies showing that ocean acidification is a major threat to crustose coralline algae (69, 70) but also suggest a compensatory effect of warming that may offset that threat, because these rhodophytes did significantly better under future ocean conditions than under Control conditions in this experiment. Overall, our mesocosm results show similar trends of decreased species diversity with selection for taxa with specific tolerances to acidification as found in previous work along natural CO_2_ gradient seeps. However, the reversal of those trends in dual-stressor future ocean conditions highlights the fact that studies from individual species exposed to single stressors are unlikely to scale predictably to ecosystem responses under combined stressors.

Coral reef ecosystems harbor highly diverse species assemblages, but the majority of research on the impacts of climate change focus on the direct and indirect effects on ecologically or economically important species, such as corals and fishes, because they are obvious and critical to ecosystem services. With over 500 million people living in proximity to coral reefs, reef fish provide subsistence, corals provide coastal protection, and together they fuel tourism that results in an annual economic value of $9.9 Trillion USD (71–73). Yet, these human-defined important species account for only a tiny fraction of the total species richness on coral reefs, and they rely critically upon the highly diverse and hidden cryptic community to sustain ecosystem function (43, 46). Over 90% of known coral reef species belong to the cryptobenthic community living within the carbonate framework (74), with an estimated 1 to 2 million remaining to be discovered (41, 75). Our experimental treatments had little effect on the persistence of corals and fishes in the mesocosms, with the major alterations of biodiversity being observed among the understudied cryptobiotic communities. The predicted loss of future reef biodiversity is based on a reduction in habitat complexity from climate change–induced coral collapse as has been shown for reef fish and a few macroinvertebrates (5, 76). However, cryptobiota diversity and densities can actually be greater under degraded frameworks (77, 78). Because these small organisms require fewer resources per individual than larger ones, they can partition resources more finely across heterogeneous microhabitats (79–82), and as a result, these communities may continue to thrive under future warming and acidification, supporting and sustaining various ecosystem processes such as nutrient release and uptake. Thus, the ultimate outcome of species shuffling in response to climate change will depend critically on the ecological roles that winners and losers play in these diverse communities, because differences in community composition and structure can alter ecosystem function (83, 84). While the ecological roles of sessile phyla (ex. Porifera, Bryozoa, Rhodophyta, and subphylum Tunicata) are relatively robust regardless of taxonomic classification, the same is not true for motile phyla that have species with fundamentally different ecological functions, even within the same families (85, 86). Until the scientific community increases functional, taxonomic, and barcoding efforts on these lesser-known taxa, identifying the species that dominate under shifting conditions and understanding their ecological functions remains a critical limitation.

Our experiments show that communities shuffle, but that biodiversity does not decline under moderate levels of ocean-warming with acidification (+2 °C, −0.2 pH). While overall richness under future ocean conditions may be similar to present-day conditions, how communities function will undoubtedly be different due to the variation in community structure and changes in organismal physiology under differing pH and temperature (84). Biodiversity is thought to be a cornerstone of reef resilience, because it may provide insurance against ecological uncertainty through functional redundancy (87, 88), and yet, we lack sufficient information on the ecological functions, life histories, and distributions (let alone names) for most members of the coral reef cryptobenthic community to be able to adequately predict responses or ecosystem outcomes of changes in this community. This critical knowledge gap in the species composition and ecological function of cryptofauna limits our ability to examine diversity–function relationships and prevents us from predicting the outcomes of community shuffling on coral reef ecosystems in response to climate change.

## Materials and Methods

### Mesocosm System.

A 40-tank outdoor, flow-through mesocosm system at the Hawai‘i Institute of Marine Biology on Coconut Island in Kāne‘ohe Bay was used to maintain experimental treatments. Unfiltered seawater pumped directly from the adjacent coral reef slope fed the fully factorial design with four treatments consisting of present-day versus end-of-century temperature and pH conditions with 10 mesocosms per treatment—refer to ref. 89 and *SI Appendix* for additional details.

### ARMS.

A total of 24 modified ARMS units (38), composed of three gray-type 1 polyvinyl chloride (PVC) plates (23 × 23 cm) forming a stacked tier of both an open and semienclosed layer, were placed individually in each of six randomly selected mesocosms (0.5 × 0.5 × 0.3 m, ∼70 L) per treatment on July 6, 2016 and removed 2 y later during the week of June 11, 2018. Upon recovery, plates were photographed, and small subsamples were collected from unique morphospecies for subsequent DNA barcoding to support the metabarcoding annotations. When subsampling was complete, each unit was individually scraped clear of all accumulated biomass which was immediately homogenized with 10 g of the homogenate subsampled and preserved in 95% ethanol for COI metabarcoding (90). One ARMS unit each from the Control treatment and the Heated treatment were accidentally contaminated during field processing. Therefore, these two units were excluded from further analyses and the remaining 22 of the original 24 ARMS units underwent the sequencing process for metabarcoding.

### DNA Metabarcoding.

Total genomic DNA from each ARMS homogenate was isolated using Powermax Soil following modifications as per Ransome et al. (91). Amplicons were generated via PCR in triplicate 20-µl reaction volumes for each sample targeting a 313-bp COI fragment using the primers mlCOIintF and jgHC02198 (92, 93) following Leray and Knowlton (90). Illumina adapters were ligated to cleaned PCR products following the Kapa Hyper-Prep Kit PCR-free protocol and sequenced at the University of California, Riverside Institute for Integrative Genome Biology (MiSeq version 3 2 × 300 bp PE).

Resulting sequences went through the bioinformatics pipeline Just Another Metabarcoding Pipeline and were clustered at 97% identity. Only MOTUs with a read abundance above 0.01% in at least one sample were considered in downstream analysis to reduce the number of false positives due to PCR and sequencing errors (94–96).

Sequences were annotated using three approaches: BLASTn against the Mo’orea BIOCODE Inventory, ecotag (97), and Informatic Sequence Classification Trees (98) to maximize annotations due to the paucity of marine invertebrate barcodes within reference databases. Only sequences annotated to metazoans and macroalgae were translated into amino acids and aligned to the BIOCODE reference data set using Multiple Alignment of Coding Sequences (MACSE) (99). Any MOTUs that did not pass through MACSE were removed (*SI Appendix*).

### Statistical Analysis.

Data were analyzed and graphed using R version 3.5.2 (R Core Team 2018). To control for differences in the numbers of sequences per library (100, 101), treatments were subsampled to an even sequencing depth. Richness was examined using a two-way ANOVA with temperature and pH as fixed factors and header tank as a nested factor followed by a post hoc Tukey pairwise comparison. Variation in community composition, defined as MOTU present/absent, and variation in community structure, defined as the relative abundance of sequencing reads, were analyzed with temperature, acidification, and their interaction within a permutational ANOVA using Jaccard and Bray–Curtis dissimilarity indices, respectively. Community data were visualized using a principal coordinate analysis. A permutational analysis of multivariate dispersion was performed to examine community dispersion. The relative read abundance of the top seven phyla and the top eight families were examined using a two-way permutational ANOVA with temperature and pH as fixed factors (*SI Appendix*).

## Supplementary Material

Supplementary File

## Data Availability

Sequencing data have been deposited in National Center for Biotechnology Information (NCBI) Sequence Read Archive (SRA): SRS7105074–SRS7105095. Data and code used in this manuscript are available at https://github.com/CuriousFauna/Cryptobiota_Climate_Change.

## References

[1] W. W. L. Cheung., Projecting global marine biodiversity impacts under climate change scenarios. Fish Fish. 10, 235–251 (2009).

[2] O. Hoegh-Guldberg., Coral reefs under rapid climate change and ocean acidification. Science 318, 1737–1742 (2007). 18079392 10.1126/science.1152509

[3] K. R. N. Anthony., Ocean acidification and warming will lower coral reef resilience: CO_2_ and coral reef resilience. Glob. Change Biol. 17, 1798–1808 (2011).

[4] J. M. Pandolfi, S. R. Connolly, D. J. Marshall, A. L. Cohen, Projecting coral reef futures under global warming and ocean acidification. Science 333, 418–422 (2011). 21778392 10.1126/science.1204794

[5] K. E. Fabricius, G. De’ath, S. Noonan, S. Uthicke, Ecological effects of ocean acidification and habitat complexity on reef-associated macroinvertebrate communities. Proc. Biol. Sci. 281, 20132479 (2013). 24307670 10.1098/rspb.2013.2479 PMC3866403

[6] J. M. Sunday., Ocean acidification can mediate biodiversity shifts by changing biogenic habitat. Nat. Clim. Chang. 7, 81–85 (2017).

[7] T. P. Hughes., Global warming and recurrent mass bleaching of corals. Nature 543, 373–377 (2017). 28300113 10.1038/nature21707

[8] C. M. Eakin, H. P. A. Sweatman, R. E. Brainard, The 2014–2017 global-scale coral bleaching event: Insights and impacts. Coral Reefs 38, 539–545 (2019).

[9] J. M. T. Magel, J. H. R. Burns, R. D. Gates, J. K. Baum, Effects of bleaching-associated mass coral mortality on reef structural complexity across a gradient of local disturbance. Sci. Rep. 9, 2512 (2019). 30792432 10.1038/s41598-018-37713-1 PMC6385266

[10] N. A. J. Graham, Habitat complexity: Coral structural loss leads to fisheries declines. Curr. Biol. 24, R359–R361 (2014). 24801184 10.1016/j.cub.2014.03.069

[11] A. Kubicek, B. Breckling, O. Hoegh-Guldberg, H. Reuter, Climate change drives trait-shifts in coral reef communities. Sci. Rep. 9, 3721 (2019). 30842480 10.1038/s41598-019-38962-4 PMC6403357

[12] K. E. Fabricius., Losers and winners in coral reefs acclimatized to elevated carbon dioxide concentrations. Nat. Clim. Chang. 1, 165–169 (2011).

[13] I. C. Enochs., Shift from coral to macroalgae dominance on a volcanically acidified reef. Nat. Clim. Chang. 5, 1083–1088 (2015).

[14] H. C. Barkley., Changes in coral reef communities across a natural gradient in seawater pH. Sci. Adv. 1, e1500328 (2015). 26601203 10.1126/sciadv.1500328 PMC4640615

[15] S. H. C. Noonan, A. Kluibenschedl, K. E. Fabricius, Ocean acidification alters early successional coral reef communities and their rates of community metabolism. PLoS One 13, e0197130 (2018). 29847575 10.1371/journal.pone.0197130 PMC5976151

[16] Y.-M. Bozec, P. J. Mumby, Synergistic impacts of global warming on the resilience of coral reefs. Philos. Trans. R. Soc. Lond. B Biol. Sci. 370, 20130267 (2015).

[17] D. P. Manzello., Poorly cemented coral reefs of the eastern tropical Pacific: Possible insights into reef development in a high-CO2 world. Proc. Natl. Acad. Sci. U.S.A. 105, 10450–10455 (2008). 18663220 10.1073/pnas.0712167105 PMC2492517

[18] P. Descombes., Forecasted coral reef decline in marine biodiversity hotspots under climate change. Glob. Change Biol. 21, 2479–2487 (2015). 10.1111/gcb.12868 25611594

[19] E. V. Kennedy., Avoiding coral reef functional collapse requires local and global action. Curr. Biol. 23, 912–918 (2013). 23664976 10.1016/j.cub.2013.04.020

[20] M. Byrne, R. Przeslawski, Multistressor impacts of warming and acidification of the ocean on marine invertebrates’ life histories. Integr. Comp. Biol. 53, 582–596 (2013). 23697893 10.1093/icb/ict049

[21] B. P. Harvey, D. Gwynn-Jones, P. J. Moore, Meta-analysis reveals complex marine biological responses to the interactive effects of ocean acidification and warming. Ecol. Evol. 3, 1016–1030 (2013). 23610641 10.1002/ece3.516 PMC3631411

[22] K. J. Kroeker., Impacts of ocean acidification on marine organisms: Quantifying sensitivities and interaction with warming. Glob. Change Biol. 19, 1884–1896 (2013). 10.1111/gcb.12179 PMC3664023 23505245

[23] R. Przeslawski, M. Byrne, C. Mellin, A review and meta-analysis of the effects of multiple abiotic stressors on marine embryos and larvae. Glob. Change Biol. 21, 2122–2140 (2015). 10.1111/gcb.12833 25488061

[24] E. D. Crook, A. L. Cohen, M. Rebolledo-Vieyra, L. Hernandez, A. Paytan, Reduced calcification and lack of acclimatization by coral colonies growing in areas of persistent natural acidification. Proc. Natl. Acad. Sci. U.S.A. 110, 11044–11049 (2013). 23776217 10.1073/pnas.1301589110 PMC3703990

[25] J. B. Ries, A. L. Cohen, D. C. McCorkle, Marine calcifiers exhibit mixed responses to CO_2_-induced ocean acidification. Geology 37, 1131–1134 (2009).

[26] M. Byrne, Impact of ocean warming and ocean acidification on marine invertebrate life history stages—Vulnerabilities and potential for persistence in a changing ocean. Oceanogr. Mar. Biol. 49, 1–42 (2011).

[27] J. A. Godbold, M. Solan, Long-term effects of warming and ocean acidification are modified by seasonal variation in species responses and environmental conditions. Philos. Trans. R. Soc. Lond. B Biol. Sci. 368, 20130186 (2013). 23980249 10.1098/rstb.2013.0186 PMC3758180

[28] N. N. Price, T. R. Martz, R. E. Brainard, J. E. Smith, Diel variability in seawater pH relates to calcification and benthic community structure on coral reefs. PLoS One 7, e43843 (2012). 22952785 10.1371/journal.pone.0043843 PMC3429504

[29] S. S. Doo., The challenges of detecting and attributing ocean acidification impacts on marine ecosystems. ICES J. Mar. Sci. 77, 2411–2422 (2020).

[30] J. S. Eklöf., Experimental climate change weakens the insurance effect of biodiversity. Ecol. Lett. 15, 864–872 (2012). 22676312 10.1111/j.1461-0248.2012.01810.x

[31] C. Alsterberg, J. S. Eklöf, L. Gamfeldt, J. N. Havenhand, K. Sundbäck, Consumers mediate the effects of experimental ocean acidification and warming on primary producers. Proc. Natl. Acad. Sci. U.S.A. 110, 8603–8608 (2013). 23630263 10.1073/pnas.1303797110 PMC3666745

[32] E. Sampaio, I. F. Rodil, F. Vaz-Pinto, A. Fernández, F. Arenas, Interaction strength between different grazers and macroalgae mediated by ocean acidification over warming gradients. Mar. Environ. Res. 125, 25–33 (2017). 28088495 10.1016/j.marenvres.2017.01.001

[33] L. Cominassi., Food availability modulates the combined effects of ocean acidification and warming on fish growth. Sci. Rep. 10, 2338 (2020). 32047178 10.1038/s41598-020-58846-2 PMC7012865

[34] B. Gaylord., Ocean acidification through the lens of ecological theory. Ecology 96, 3–15 (2015). 26236884 10.1890/14-0802.1

[35] M. Solan., Extinction and ecosystem function in the marine benthos. Science 306, 1177–1180 (2004). 15539601 10.1126/science.1103960

[36] D. Lawrence., Species interactions alter evolutionary responses to a novel environment. PLoS Biol. 10, e1001330 (2012). 22615541 10.1371/journal.pbio.1001330 PMC3352820

[37] J. M. Alexander, J. M. Diez, S. P. Hart, J. M. Levine, When climate reshuffles competitors: A call for experimental macroecology. Trends Ecol. Evol. 31, 831–841 (2016). 27640784 10.1016/j.tree.2016.08.003 PMC5159619

[38] M. A. Timmers, J. Vicente, M. Webb, C. P. Jury, R. J. Toonen, Sponging up diversity: Evaluating metabarcoding performance for a taxonomically challenging phylum within a complex cryptobenthic community. Environ DNA10.1002/edn3.163 (2020).

[39] J. Rogelj., Paris Agreement climate proposals need a boost to keep warming well below 2 °C. Nature 534, 631–639 (2016). 27357792 10.1038/nature18307

[40] K. D. Gorospe., Local biomass baselines and the recovery potential for Hawaiian coral reef fish communities. Front. Mar. Sci. 5, 162 (2018).

[41] M. L. Reaka-Kudla, “The global biodiversity of coral reefs: A comparison with rain forests” in Biodiversity II: Understanding and Protecting Our Natural Resources, M. L. Reaka-Kudla, D. E. Wilson, E. O. Eilson, Eds. (Joseph Henry/National Academy Press, 1997), pp. 83–108.

[42] J. L. Wulff, Sponge-mediated coral reef growth and rejuvenation. Coral Reefs 3, 157–163 (1984).

[43] J. M. de Goeij., Surviving in a marine desert: The sponge loop retains resources within coral reefs. Science 342, 108–110 (2013). 24092742 10.1126/science.1241981

[44] J. L. Wulff, L. W. Buss, Do sponges help hold coral reefs together? Nature 281, 474–475 (1979).

[45] K. Wolfe., Current and future trophic interactions in tropical shallow-reef lagoon habitats. Coral Reefs 40, 83–96 (2021).

[46] S. J. Brandl., Demographic dynamics of the smallest marine vertebrates fuel coral reef ecosystem functioning. Science 364, 1189–1192 (2019). 31123105 10.1126/science.aav3384

[47] N. Vogel., Interactive effects of ocean acidification and warming on coral reef associated epilithic algal communities under past, present-day and future ocean conditions. Coral Reefs 35, 715–728 (2016).

[48] F. Prada., Ocean warming and acidification synergistically increase coral mortality. Sci. Rep. 7, 40842 (2017). 28102293 10.1038/srep40842 PMC5244398

[49] K. J. Kroeker., The role of temperature in determining species’ vulnerability to ocean acidification: A case study using *Mytilus galloprovincialis*. PLoS One 9, e100353 (2014). 24984016 10.1371/journal.pone.0100353 PMC4077567

[50] C. P. Jury, R. J. Toonen, Adaptive responses and local stressor mitigation drive coral resilience in warmer, more acidic oceans. Proc. Biol. Sci. 286, 20190614 (2019). 31088274 10.1098/rspb.2019.0614 PMC6532518

[51] J.-H. Kim., Global warming offsets the ecophysiological stress of ocean acidification on temperate crustose coralline algae. Mar. Pollut. Bull. 157, 111324 (2020). 32658689 10.1016/j.marpolbul.2020.111324

[52] J. Garzke, T. Hansen, S. M. H. Ismar, U. Sommer, Combined effects of ocean warming and acidification on copepod abundance, body size and fatty acid content. PLoS One 11, e0155952 (2016). 27224476 10.1371/journal.pone.0155952 PMC4880321

[53] K. A. Pitts, J. E. Campbell, J. Figueiredo, N. D. Fogarty, Ocean acidification partially mitigates the negative effects of warming on the recruitment of the coral, *Orbicella faveolata*. Coral Reefs 39, 281–292 (2020).

[54] S. S. Doo, R. C. Carpenter, P. J. Edmunds, Obligate ectosymbionts increase the physiological resilience of a scleractinian coral to high temperature and elevated pCO_2_. Coral Reefs 37, 997–1001 (2018).

[55] S. S. Doo., Amelioration of ocean acidification and warming effects through physiological buffering of a macroalgae. Ecol. Evol. 10, 8465–8475 (2020). 32788994 10.1002/ece3.6552 PMC7417211

[56] G. Ghedini, B. D. Russell, S. D. Connell, Trophic compensation reinforces resistance: Herbivory absorbs the increasing effects of multiple disturbances. Ecol. Lett. 18, 182–187 (2015). 25581377 10.1111/ele.12405

[57] S. Dupont, J. Havenhand, W. Thorndyke, L. Peck, M. Thorndyke, Near-future level of CO_2_-driven ocean acidification radically affects larval survival and development in the brittlestar *Ophiothrix fragilis*. Mar. Ecol. Prog. Ser. 373, 285–294 (2008).

[58] N. Espinel-Velasco., Effects of ocean acidification on the settlement and metamorphosis of marine invertebrate and fish larvae: A review. Mar. Ecol. Prog. Ser. 606, 237–257 (2018).

[59] J. M. Leis, Paradigm lost: Ocean acidification will overturn the concept of larval-fish biophysical dispersal. Front. Mar. Sci. 5, 1–9 (2018).29552559

[60] K. S. Nelson, F. Baltar, M. D. Lamare, S. E. Morales, Ocean acidification affects microbial community and invertebrate settlement on biofilms. Sci. Rep. 10, 3274 (2020). 32094391 10.1038/s41598-020-60023-4 PMC7039980

[61] N. S. Webster, S. Uthicke, E. S. Botté, F. Flores, A. P. Negri, Ocean acidification reduces induction of coral settlement by crustose coralline algae. Glob. Change Biol. 19, 303–315 (2013). 10.1111/gcb.12008 PMC3597258 23504741

[62] G. V. Ashton, S. A. Morley, D. K. A. Barnes, M. S. Clark, L. S. Peck, Warming by 1°C drives species and assemblage level responses in Antarctica’s marine shallows. Curr. Biol. 27, 2698–2705.e3 (2017). 28867203 10.1016/j.cub.2017.07.048

[63] K. J. Kroeker, F. Micheli, M. C. Gambi, Ocean acidification causes ecosystem shifts via altered competitive interactions. Nat. Clim. Chang. 3, 156–159 (2013).

[64] E. D. Crook., Recruitment and succession in a tropical benthic community in response to in-situ ocean acidification. PLoS One 11, e0146707 (2016). 26784986 10.1371/journal.pone.0146707 PMC4718464

[65] P. Mladenov, R. Emson, Density, size structure and reproductive characteristics of fissiparous brittle stars in algae and sponges: Evidence for interpopulational variation in levels of sexual and asexual reproduction. Mar. Ecol. Prog. Ser. 42, 181–194 (1988).

[66] S. Stöhr, T. D. O’Hara, B. Thuy, Global diversity of brittle stars (Echinodermata: Ophiuroidea). PLoS One 7, e31940 (2012). 22396744 10.1371/journal.pone.0031940 PMC3292557

[67] N. M. Lucey., To brood or not to brood: Are marine invertebrates that protect their offspring more resilient to ocean acidification? Sci. Rep. 5, 12009 (2015). 26156262 10.1038/srep12009 PMC4648422

[68] K. J. Kroeker, F. Micheli, M. C. Gambi, T. R. Martz, Divergent ecosystem responses within a benthic marine community to ocean acidification. Proc. Natl. Acad. Sci. U.S.A. 108, 14515–14520 (2011). 21844331 10.1073/pnas.1107789108 PMC3167536

[69] I. B. Kuffner, A. J. Andersson, P. L. Jokiel, K. S. Rodgers, F. T. Mackenzie, Decreased abundance of crustose coralline algae due to ocean acidification. Nat. Geosci. 1, 114–117 (2008).

[70] C. E. Cornwall, G. Diaz-Pulido, S. Comeau, Impacts of ocean warming on coralline algal calcification: Meta-analysis, knowledge gaps, and key recommendations for future research. Front. Mar. Sci. 6, 1–10 (2019).

[71] D. Bryant, L. Burke, J. MacManus, M. Spalding, Reefs at Risk: A Map-Based Indicator of Threats to the World’s Coral Reefs (World Resources Institute, 1998).

[72] C. Wilkinson, Status of Coral Reefs of the World (Reef and Rain Forest Research Centre, 2008).

[73] R. Costanza., Changes in the global value of ecosystem services. Glob. Environ. Change 26, 152–158 (2014).

[74] J. Stella, M. Pratchett, P. Hutchings, G. Jones, Coral-associated invertebrates: Diversity, ecological importance and vulnerability to disturbance. Oceanogr. Mar. Biol. 49, 43–104 (2011).

[75] R. Fisher., Species richness on coral reefs and the pursuit of convergent global estimates. Curr. Biol. 25, 500–505 (2015). 25639239 10.1016/j.cub.2014.12.022

[76] N. A. J. Graham., Dynamic fragility of oceanic coral reef ecosystems. Proc. Natl. Acad. Sci. U.S.A. 103, 8425–8429 (2006). 16709673 10.1073/pnas.0600693103 PMC1482508

[77] I. Enochs, L. Toth, V. Brandtneris, J. Afflerbach, D. Manzello, Environmental determinants of motile cryptofauna on an eastern Pacific coral reef. Mar. Ecol. Prog. Ser. 438, 105–118 (2011).

[78] I. C. Enochs, D. P. Manzello, Species richness of motile cryptofauna across a gradient of reef framework erosion. Coral Reefs 31, 653–661 (2012).

[79] A. J. Kohn, P. J. Leviten, Effect of habitat complexity on population density and species richness in tropical intertidal predatory gastropod assemblages. Oecologia 25, 199–210 (1976). 28308866 10.1007/BF00345098

[80] E. M. Sides, D. Woodley, Niche separation in the three species of ophiocoma (Echinodermata Ophiurodea) in Jamaica, West Indies. Bull. Mar. Sci. 36, 701–715 (1985).

[81] D. Moran, M. Reaka, Bioerosion and availability of shelter for benthic reef organisms. Mar. Ecol. Prog. Ser. 44, 249–263 (1988).

[82] J. Tews., Animal species diversity driven by habitat heterogeneity/diversity: The importance of keystone structures. J. Biogeogr. 31, 79–92 (2004).

[83] M. L. Avolio., Changes in plant community composition, not diversity, during a decade of nitrogen and phosphorus additions drive above-ground productivity in a tallgrass prairie. J. Ecol. 102, 1649–1660 (2014).

[84] N. J. Silbiger, C. J. B. Sorte, Biophysical feedbacks mediate carbonate chemistry in coastal ecosystems across spatiotemporal gradients. Sci. Rep. 8, 796 (2018). 29335493 10.1038/s41598-017-18736-6 PMC5768679

[85] P. A. Jumars, K. M. Dorgan, S. M. Lindsay, Diet of worms emended: An update of polychaete feeding guilds. Annu. Rev. Mar. Sci. 7, 497–520 (2015). 10.1146/annurev-marine-010814-020007 25251269

[86] A. Kohn, “Feeding biology of gastropods” in The Mollusca, A. S. M. Saleuddin, K. M. Wilbur, Eds. (Academic Press, 1983), 5, pp. 1–63.

[87] M. Nyström, Redundancy and response diversity of functional groups: Implications for the resilience of coral reefs. AMBIO 35, 30–35 (2006). 16615697

[88] S. J. Brandl., Coral reef ecosystem functioning: Eight core processes and the role of biodiversity. Front. Ecol. Environ. 17, 445–454 (2019).

[89] K. D. Bahr, T. Tran, C. P. Jury, R. J. Toonen, Abundance, size, and survival of recruits of the reef coral *Pocillopora acuta* under ocean warming and acidification. PLoS One 15, e0228168 (2020). 32017776 10.1371/journal.pone.0228168 PMC6999881

[90] M. Leray, N. Knowlton, DNA barcoding and metabarcoding of standardized samples reveal patterns of marine benthic diversity. Proc. Natl. Acad. Sci. U.S.A. 112, 2076–2081 (2015). 25646458 10.1073/pnas.1424997112 PMC4343139

[91] E. Ransome., The importance of standardization for biodiversity comparisons: A case study using autonomous reef monitoring structures (ARMS) and metabarcoding to measure cryptic diversity on Mo’orea coral reefs, French Polynesia. PLoS One 12, e0175066 (2017). 28430780 10.1371/journal.pone.0175066 PMC5400227

[92] J. Geller, C. Meyer, M. Parker, H. Hawk, Redesign of PCR primers for mitochondrial cytochrome c oxidase subunit I for marine invertebrates and application in all-taxa biotic surveys. Mol. Ecol. Resour. 13, 851–861 (2013). 23848937 10.1111/1755-0998.12138

[93] M. Leray., A new versatile primer set targeting a short fragment of the mitochondrial COI region for metabarcoding metazoan diversity: Application for characterizing coral reef fish gut contents. Front. Zool. 10, 34 (2013). 23767809 10.1186/1742-9994-10-34 PMC3686579

[94] N. A. Bokulich., Quality-filtering vastly improves diversity estimates from Illumina amplicon sequencing. Nat. Methods 10, 57–59 (2013). 23202435 10.1038/nmeth.2276 PMC3531572

[95] V. Elbrecht, F. Leese, Validation and development of COI metabarcoding primers for freshwater macroinvertebrate bioassessment. Front. Environ. Sci. 5, 11 (2017).

[96] I. Bista., Annual time-series analysis of aqueous eDNA reveals ecologically relevant dynamics of lake ecosystem biodiversity. Nat. Commun. 8, 14087 (2017). 28098255 10.1038/ncomms14087 PMC5253663

[97] F. Boyer., obitools: A unix-inspired software package for DNA metabarcoding. Mol. Ecol. Resour. 16, 176–182 (2016). 25959493 10.1111/1755-0998.12428

[98] S. P. Wilkinson, S. K. Davy, M. Bunce, M. Stat, Taxonomic identification of environmental DNA with informatic sequence classification trees. PeerJ Preprints [Preprint] (2018). 10.7287/peerj.preprints.26812v1 (Accessed 24 November 2020).

[99] V. Ranwez, S. Harispe, F. Delsuc, E. J. P. Douzery, MACSE: Multiple Alignment of Coding SEquences accounting for frameshifts and stop codons. PLoS One 6, e22594 (2011). 21949676 10.1371/journal.pone.0022594 PMC3174933

[100] N. J. Gotelli, R. K. Colwell, Quantifying biodiversity: Procedures and pitfalls in the measurement and comparison of species richness. Ecol. Lett. 4, 379–391 (2001).

[101] S. Weiss., Normalization and microbial differential abundance strategies depend upon data characteristics. Microbiome 5, 27 (2017). 28253908 10.1186/s40168-017-0237-y PMC5335496

