# Supplementary material for: Biodiversity of coral reef cryptobiota shuffles but does not decline under the combined stressors of ocean warming and acidification
Source: Proc Natl Acad Sci U S A. 2021 Sep 20;118(39):e2103275118. doi: 10.1073/pnas.2103275118 (PMC8488634; doi:10.1073/pnas.2103275118)
Supplement: Supplementary File [file pnas.2103275118.sapp.pdf]

PNAS
www.pnas.org

Supplementary Information for

Biodiversity of coral reef cryptobiota shuffles but does not decline under the combined stressors of ocean warming and acidification

Molly A. Timmers[1-3], Christopher P. Jury[3], Jan Vicente[3], Keisha D. Bahr[4], Maryann K. Webb[3], Robert J. Toonen[3]

Molly A. Timmers
Email: timmers@hawaii.edu

This PDF file includes:

- Supplementary Text
- SI References
- Figures S1 to S10
- Tables S1 to S14

Supplementary Text

Detailed Tank Set-up:

A 40-tank outdoor, flow-through mesocosm system at the Hawai‘i Institute of Marine Biology on Coconut Island in Kāne‘ohe Bay was used to maintain experimental treatments. Unfiltered seawater pumped continuously and directly from the adjacent coral reef slope fed the fully factorial design with four treatments consisting of Control present-day and future ocean temperature and pH conditions (10 mesocosms per treatment) (Figure S1). Relative to the offshore source water, seawater temperature and chemistry are naturally modified by reef-associated physical and biogeochemical processes as the water flows through the bay. To ensure that the water temperature and chemistry of the incoming seawater was close to that of the original source water rather than altered by the reef-associated physical and biogeochemical processes, all incoming seawater was directed into a mixing tank where temperature was adjusted slightly using a commercial heat pump on a temperature controller and chemistry was marginally adjusted with small additions of 1.0 N NaOH via a peristaltic pump to achieve average present-day offshore temperature and chemistry conditions in Hawai‘i (temperature ~23.5–27.5 °C, pH ~7.97–8.07, annually). These small adjustments resulted in modifications to the temperature, pH, and total alkalinity in the seawater input which, when combined with the same physical and biogeochemical processes in the mesocosms, allowed us to achieve conditions similar to those observed on the reef (1–3) and expose all in-coming larvae to the same environment prior to treatment conditioning. The water from this mixing tank was then split off into a series of header tanks where it was heated or acidified according to treatment, with 2 replicate header tanks per treatment to avoid pseudoreplication. Temperature was adjusted using commercial aquarium heaters on temperature controllers and seawater was dosed with CO_2 gas using high precision needle valves connected to venturi valves on aquarium pumps to deliver a precisely controlled quantity of CO_2 gas that was completely dissolved into the seawater in the header tanks before flowing into the mesocosms. The Heated treatments were set to remain 2 °C above the Control (i.e., present-day average) temperature, whereas the Acidified treatments were maintained at 0.2 pH units below the Control treatment, thereby replicating conditions expected at the end of the century. From the header tanks, the treated water was distributed to the respective mesocosm treatments. Water flowed continuously into each mesocosm at an inflow rate at 1.2 L min^{-1} providing a 1 hr residence time within all mesocosms. Additional water circulation was generated within each mesocosm a Maxi-Jet Pro propeller (4900 L hr^{-1}) seawater pump. All mesocosms experienced natural daily and seasonal fluctuations in light, seawater temperature, and carbonate chemistry with appropriate offsets according to treatment.

Two approaches were used to characterize the water temperature and chemistry in the mesocosms. First, water samples were taken from each mesocosm at 1200 hr local time once per week for total alkalinity and spectrophotometric pH, whereas salinity and temperature were measured with a YSI multimeter. All these procedures followed standard protocols (4). The precisions of these measurements were: pH ±0.002 units, salinity ±0.01 psu, temperature ±0.01 °C, total alkalinity ±7 μmol kg^{-1}. The accuracies of

these measurements are estimated as: pH ±0.005 units, salinity ±0.3 psu, temperature ±0.1 °C, total alkalinity ±7 µmol kg^{-1}. The accuracy and precision of total alkalinity titrations were assessed using Certified Reference Materials (CRMs) obtained from Andrew Dickson (Scripps Institution of Oceanography). Second, the temperature and chemistry measurements as described above, were assessed every 4 hr over the diel cycle once per quarter. For the remaining two months per quarter, bottle samples were taken for pH and total alkalinity only at 1200 and 0000 hr and a pH meter was used to assess pH at the other diel sampling points (1600, 2000, 0400, and 0800 hr). The pH meter was empirically calibrated to the 1200 and 0000 hr pH bottle samples at the time of collection, yielding an uncertainty of ±0.02 units for these sample points. The monthly diel samples were used to assess the hourly temperature and chemistry variation in the mesocosms as well as the daily mean values on those sampling dates. To estimate the daily mean values from the weekly water samples, the empirically derived relationships between the offset of the mesocosm sea water to the incoming sea water measured at 1200 hr and the daily mean values (characterized during the diel sampling) were used to estimate the daily mean parameters. These estimates yielded the following uncertainties in the calculated daily means: salinity ±0.12 psu, pH ±0.03 units, temperature ±0.17 °C, and total alkalinity ±16 µmol kg^{-1}. The remaining carbonate chemistry parameters were calculated with CO2SYS (5).

To simulate a reef environment, each mesocosm included coral nubbins from the eight-dominant reef-building coral species in Hawaii (*Montipora capitata, Montipora flabellata, Montipora patula, Pocillopora acuta, Pocillopora meandrina, Porites compressa, Porites evermanni,* and *Porites lobata*), yielding an initial coral cover of roughly 10%. These coral species collectively comprise >95% of the coral cover on Hawaiian reefs (6, 7). Five herbivorous reef snails (*Trochus sp.),* a juvenile Threadfin butterflyfish (*Chaetodon auriga*), and a juvenile Convict tang (*Acanthurus triostegus*) were placed in each mesocosm. The surgeonfish is a generalist grazer on benthic algae whereas the butterflyfish is a generalist grazer on non-coral invertebrates. Together, the fish provided the essential ecological functions of herbivory and predation in the mesocosms, and at fish biomass values similar to those reported for Hawaiian reefs (8). Each mesocosm had a 2 cm layer of carbonate reef sand and gravel and pieces of reef rubble (3 replicate 10-20 cm pieces). All visible organisms were removed from the sand, gravel, and rubble which were then well mixed and randomly divided among mesocosms to avoid any sort of bias among treatments. The corals and rubble were placed on a plastic grate 5 cm above the reef sand and gravel to simulate their attachment to hard substrate in nature.

This mesocosm community was given 12 weeks to acclimate under ambient flow-through sea water before slowly adjusting the temperature and pH over the course of 20 days. Four months later, the ARMS were placed underneath the plastic grate to simulate the cryptobenthos (Fig. S2).

Detailed Metabarcoding Processing

I. DNA Extraction

Total genomic DNA from each ARMS homogenate was isolated using Powermax Soil Isolation Kit following modifications to the manufacturer's protocol (9). Amplicons of the cytochrome oxidase I gene (COI) were generated via polymerase chain reaction (PCR) in triplicate 20 µl reaction volumes for each sample, targeting a 313 bp fragment using the primers mICOlintF (5′ GGW ACW GGW TGA ACW GTW TAY CCY CC 3′ (10) and jgHCO2198 (5′ TAI ACY TCI GGR TGI CCR AAR AAY CA 3′ (11). Each 20 µl reaction included: 7.65 µl of nanopure H_2O, 10 µl of ImmoMix Red (2×; Bioline), 0.06 µl of each primer (10 µM), 0.15 µl bovine serum albumin (10 mg/ml) and 1 µl template DNA (5–25 ng μl^{-1}). We used a touchdown PCR profile with 16 initial cycles: denaturation for 10 s at 95 °C, annealing for 30 s at 62 °C (-1 °C per cycle), and extension for 60 s at 72 °C, followed by 20 cycles at an annealing temperature of 46 °C (10). All PCR reactions included negative controls and PCR products were quality assessed by gel electrophoresis in agarose gel. Amplification success was defined by the presence of a clear band around 325 bp. If a band was encountered in the negative control, all associated PCR products were discarded and redone. Pooled triplicate reactions along with the negative controls were purified with Agencourt AMPure XP beads and quality assessed again by gel electrophoresis. Illumina adapters were ligated to cleaned PCR products using the Kapa Hyper-Prep PCR-free Kit and assessed by gel electrophoresis. If any of the negative controls had visible bands >100 bp, all associated samples were discarded and the PCR process and library preparation were reinitiated. Libraries were validated via qPCR using the KAPA library quantification kit and sized and checked for quality using an Agilent Technologies 2100 Bioanalyzer. Samples passing quality control were sequenced at the University of California, Riverside's Institute for Integrative Genome Biology on MiSeq platforms using v3 chemistry (2 × 300 bp paired-ends).

II. Bioinformatics

We used the metabarcoding R modular package pipeline *Just Another Metabarcoding Pipeline* (JAMP - https://github.com/VascoElbrecht/JAMP) which integrates Usearch v10.0.240 (12), Vsearch v2.4.3 (13) and Cutadapt 1.9 (14) to process all samples. In brief, preprocessing of reads included sample demultiplexing, paired-end merging (Usearch, allowing for 25% mismatches in overlap), primer trimming (Cutadapt, allowing for 10% errors in primer matching), generation of reverse complements to align reads in the forward direction (Usearch), and sequence length filtration (min/max 295/340 bp – Cutadapt; (15)). Low quality sequences were filtered and discarded using UPARSE fastq_filter with maxee = 0.25 and qmax at 60 (16), dereplicated (min. unique size = 2), and clustered with simultaneous chimera removal using USEARCH (cluster_otus 97% identity). The pre-processed dereplicated reads of all samples (including singletons) were matched against the respective Molecular Operational Taxonomic Units (MOTUs) with a minimum match of 97% using usearch_global and strand plus within USEARCH. MOTUs with a read abundance above 0.01% in at least one sample were considered in downstream analysis to reduce the number of false positives due to PCR and sequencing errors (17–19).

III. Sequence annotation

MOTUs were classified using three approaches to maximize taxonomic assignments. We first ran a local BLASTn against 98 DNA barcodes obtained from vouchers sampled from the ARMS plates and against a curated reference database containing 16,679 COI sequences specific to coral reef fauna from the Mo'orea Biocode Inventory (http://biocode.berkeley.edu/). Second, we classified sequences using the ecotag algorithm (20), which takes a lowest common ancestor classification approach on representative sequences for each molecular operational taxonomic unit (MOTU) taxa in relation to a reference COI database (db COI Nov2018) that contained 192,929 filtered COI sequences (21). Lastly, we assigned sequences using the R package, Informatic Sequence Classification Trees (INSECT), that takes a probabilistic approach (hidden Markov model) to assignment against a classification tree built from 396,413 sequences extracted from the MIDORI database and GenBank (22).

Due to the limited number of marine invertebrate barcodes within reference databases, BLASTn identifications were accepted at ≥ 85% identity, ≥ 85 % coverage, and ≥ 200 alignment length (9). Ecotag classifications were accepted if the "best identity" was ≥ 85% and INSECT assignments were set at a probability of ≥ 0.80%. We examined assignments across competing methods using a customized R script that assigned a "Final Phylum' and "Final Kingdom" based on the following step-wise hierarchical decision tree: 1) Sequences identified by the DNA barcodes obtained from this experiment or from the curated Mo'orea Biocode Database were accepted; 2) Assignments were accepted when the two remaining methods had matching phyla level identifications; 3) Assignments were accepted by the method when no other method had an assignment to the representative MOTU; and 4) we accepted the INSECT classification if the Akaike weight score was > 0.9 and ecotag identification was < 0.90. For the few remaining outliers, assignment was based on the method with the greatest identity.

Using the Final Phylum and Final Kingdom assignments, we separated annotations into the following groups: metazoa, macroalgae, bacteria, unicellular algae, fungi, other eukaryote, other opisthokonta, and unclassified. To separate out the macroalgae and unicellular algae within the phylum Ochrophyta, MOTUs identified by ecotag and INSECT to the class Phaeophyceae were assigned to macroalgae. The macroalgae group consists of only red (Rhodophyta) and brown (Ochrophyta) algae due to the COI barcoding region being able to differentiate among species within these two algal groups (23–25). All MOTUs assigned to green algae (Chlorophyta) were removed because the COI region is not suitable for green algae (26). Within the phylum Chordata, only MOTUs assigned to the class Ascidiacea, subphylum Tunicata, were retained. Likewise, MOTUs assigned to the classes Insecta and Arachnida in the phylum Arthropoda were removed. For downstream analyses, we selected only those MOTUs assigned to the group metazoa and macroalgae which represented 77.6% of the sequences. For further classifications, we conservatively accepted Class, Order, Family, Genus, and Species annotations if percent identity was ≥ 90%, ≥ 92%, ≥ 95%, >97%, and ≥ 98%, respectively, and removed MOTUs (17.7%) that had no

taxonomic match to the phylum level. The exception being sponge species for which we excepted species identification if percent identity was 100% (15). All remaining MOTUs were translated into amino acids and aligned to the BIOCODE reference data set using Multiple Alignment of Coding Sequences (MACSE) (27). MACSE detects interruptions in open reading frames from nucleotide substitutions that can result in stop codons which are likely to be pseudogenes. Any MOTUs that did not pass through MACSE were removed. Refer to Timmers et al (15) for more details on the accuracy of metabarcoding performance in the taxonomic validation of the phyla Porifera found on these ARMS plates.

Statistical Analysis

Data were analyzed using R version 3.5.2 (R Core Team 2018). To control for the effects of library size estimates (numbers of sequences) (28, 29) and to ensure that the number of reads per MOTU were comparable across treatments, resulting community data were randomly rarefied using 100 repeated independent rarefractions using the 'rarefy.perm' function in *EcolUtils* (30). To examine unique and shared MOTUs among treatments, the overLapper function in the R package *systemPipeR* (31) was used on a random draw of 5 ARMS units per treatment to account for uneven sample sizes. The results were graphed using the 'olBarplot' function.

Observed MOTU richness was calculated using the 'specnumber' function in *vegan* (32) and analyzed using a 2-way ANOVA with temperature and pH as fixed factors nested within header tank. A post-hoc Tukey pairwise comparison was analyzed using the 'lsmeans' function in *lsmeans* (33). Assumptions of normality, equality of variance, and independence were assessed on the residuals via diagnostic plots and using shapiro.test, barlett.test, leveneTest, and the durbinWatsonTest functions from the package *car* (34).

Variation in community composition, defined as MOTU presence/absence, from temperature, acidification, and their interaction was analyzed using a Jaccard dissimilarity index within a permutational analysis of variance (PERMANOVA – 'adonis' function in *vegan*). The variation in community structure, defined as MOTU sequence relative abundance (35, 36), from temperature, acidification, and their interaction was analyzed on a Bray-Curtis distance matrix using a Hellinger standardization (square-root of relative abundance) in a PERMANOVA framework. A pairwise PERMANOVA using the 'adonis.pair' function with a false discovery adjustment in *EcolUtils* was conducted to examine differences between community composition and structure among treatments. A permutational analysis of multivariate dispersion (PERMDISP- 'betadisper' function in *vegan*) was performed on community composition and structure among replicates to examine community dispersion within treatments. A pairwise PERMDISP ('permutest.betadisper' function in *vegan*) was conducted to examine differences in variabilities among treatments and community data were visualized using a principal coordinate analysis (PCoA).

We examined the relationship of reads and MOTUs pooled to phyla among treatments relative to the Control treatment by taking a random draw of 5 ARMS units per treatment, to account for uneven sample sizes. We conducted a permutational 2-way ANOVA based on 999 permutations using the perm.anova function in *RVAideMemoire* to examine variations in the read abundance of the top 7 phyla with temperature and pH as fixed factors. We pooled taxa to the family level and classified families into the following calcification levels: heavily calcified, limited calcification, and no calcification. Taxa that have calcium carbonate skeletal forms, such as marine snails and brittle stars, were classified as heavily calcified, while limited calcification included taxa which incorporate the biomineralization of carbonate into part of their body form, such as crustaceans (37). Calcareous sponges were not included in this analysis due to the inability of the COI primers to amplify sponges from the class Calcarea (15). Those families that represented a minimum of 4% of the reads were examined using a permutational 2-way ANOVA and the relationship of reads among treatments relative to the Control treatment were examined and visualized.

SI References:

1. K. E. F. Shamberger, *et al.*, Calcification and organic production on a Hawaiian coral reef. *Mar Chem* **127**, 64–75 (2011).

2. Ò. Guadayol, N. J. Silbiger, M. J. Donahue, F. I. M. Thomas, Patterns in Temporal Variability of Temperature, Oxygen and pH along an Environmental Gradient in a Coral Reef. *PLoS ONE* **9**, e85213 (2014).

3. C. P. Jury, M. N. Delano, R. J. Toonen, High heritability of coral calcification rates and evolutionary potential under ocean acidification. *Sci Rep* **9**, 20419 (2019).

4. A. G. Dickson, C. L. Sabine, J. R. Christian, C. P. Bargeron, North Pacific Marine Science Organization, Eds., *Guide to best practices for ocean CO2 measurements* (North Pacific Marine Science Organization, 2007).

5. E. Lewis, D. W. R. Wallace, Program Developed for CO2 System Calculations, ORNL/CDIAC-105 (1998).

6. K. S. Rodgers, P. L. Jokiel, E. K. Brown, S. Hau, R. Sparks, Over a Decade of Change in Spatial and Temporal Dynamics of Hawaiian Coral Reef Communities. *Pac Sci* **69**, 1–13 (2015).

7. E. Franklin, P. Jokiel, M. Donahue, Predictive modeling of coral distribution and abundance in the Hawaiian Islands. *Mar. Ecol. Prog. Ser.* **481**, 121–132 (2013).

8. K. D. Gorospe, *et al.*, Local Biomass Baselines and the Recovery Potential for Hawaiian Coral Reef Fish Communities. *Front. Mar. Sci.* **5**, 162 (2018).

9. E. Ransome, *et al.*, The importance of standardization for biodiversity comparisons: A case study using autonomous reef monitoring structures (ARMS) and metabarcoding to measure cryptic diversity on Mo'orea coral reefs, French Polynesia. *PLoS ONE* **12**, e0175066 (2017).

10. M. Leray, *et al.*, A new versatile primer set targeting a short fragment of the mitochondrial COI region for metabarcoding metazoan diversity: application for characterizing coral reef fish gut contents. *Front Zool* **10**, 34 (2013).

11. J. Geller, C. Meyer, M. Parker, H. Hawk, Redesign of PCR primers for mitochondrial cytochrome *c* oxidase subunit I for marine invertebrates and application in all-taxa biotic surveys. *Mol Ecol Resour* **13**, 851–861 (2013).

12. R. C. Edgar, UPARSE: highly accurate OTU sequences from microbial amplicon reads. *Nat Methods* **10**, 996–998 (2013).

13. T. Rognes, T. Flouri, B. Nichols, C. Quince, F. Mahé, VSEARCH: a versatile open source tool for metagenomics. *PeerJ* **4**, e2584 (2016).

14. M. Martin, Cutadapt removes adapter sequences from high-throughput sequencing reads. *EMBnet Journal*, 10–12 (2011).

15. M. A. Timmers, J. Vicente, M. Webb, C. P. Jury, R. J. Toonen, Sponging up diversity: Evaluating metabarcoding performance for a taxonomically challenging phylum within a complex cryptobenthic community. *Environ DNA*, edn3.163 (2020).

16. R. C. Edgar, H. Flyvbjerg, Error filtering, pair assembly and error correction for next-generation sequencing reads. *Bioinformatics* **31**, 3476–3482 (2015).

17. N. A. Bokulich, *et al.*, Quality-filtering vastly improves diversity estimates from Illumina amplicon sequencing. *Nat Methods* **10**, 57–59 (2013).

18. V. Elbrecht, F. Leese, Validation and Development of COI Metabarcoding Primers for Freshwater Macroinvertebrate Bioassessment. *Front. Environ. Sci.* **5** (2017).

19. I. Bista, *et al.*, Annual time-series analysis of aqueous eDNA reveals ecologically relevant dynamics of lake ecosystem biodiversity. *Nat Commun* **8**, 14087 (2017).

20. F. Boyer, *et al.*, OBITOOLS: a UNIX-inspired software package for DNA metabarcoding. *Mol Ecol Resour* **16**, 176–182 (2016).

21. O. S. Wangensteen, C. Palacín, M. Guardiola, X. Turon, DNA metabarcoding of littoral hard-bottom communities: high diversity and database gaps revealed by two molecular markers. *PeerJ* **6**, e4705 (2018).

22. S. P. Wilkinson, S. K. Davy, M. Bunce, M. Stat, "Taxonomic identification of environmental DNA with informatic sequence classification trees." (PeerJ Preprints, 2018) https:/doi.org/10.7287/peerj.preprints.26812v1 (November 24, 2020).

23. D. C. McDevit, G. W. Saunders, On the utility of DNA barcoding for species differentiation among brown macroalgae (Phaeophyceae) including a novel extraction protocol. *Phycol Res* **57**, 131–141 (2009).

24. L. Le Gall, G. W. Saunders, DNA barcoding is a powerful tool to uncover algal diversity: a case study of the Phyllophoraceae (Gigartinales, Rhodophyta) in the Canadian flora. *J Phycol* **46**, 374–389 (2010).

25. A. R. Sherwood, A. Kurihara, K. Y. Conklin, T. Sauvage, G. G. Presting, The Hawaiian Rhodophyta Biodiversity Survey (2006-2010): a summary of principal findings. *BMC Plant Biol* **29** (2010).

26. G. W. Saunders, H. Kucera, An evaluation of rbcL, tufA, UPA, LSU and ITS as DNA barcode markers for the marine green macroalgae. *Cryptogamie Algologie* **31**, 487–528 (2010).

27. V. Ranwez, S. Harispe, F. Delsuc, E. J. P. Douzery, MACSE: Multiple Alignment of Coding SEquences Accounting for Frameshifts and Stop Codons. *PLoS ONE* **6**, e22594 (2011).

28. N. J. Gotelli, R. K. Colwell, Quantifying biodiversity: procedures and pitfalls in the measurement and comparison of species richness. *Ecol Lett* **4**, 379–391 (2001).

29. S. Weiss, *et al.*, Normalization and microbial differential abundance strategies depend upon data characteristics. *Microbiome* **5**, 27 (2017).

30. G. Salazar, *EcolUtils: Utilities for community ecology analysis* (2020).

31. T. W. H. Backman, T. Girke, systemPipeR: NGS workflow and report generation environment. *BMC Bioinformatics* **17**, 388 (2016).

32. J. Oksanen, G. Blanchel, M. Friendly, R. Kindt, P. Legendre, *vegan: Community Ecology Package* (2019).

33. R. V. Lenth, Least-Squares Means: The *R* Package lsmeans. *J. Stat. Soft.* **69** (2016).

34. J. Fox, S. Weisberg, J. Fox, *An R companion to applied regression*, 2nd ed (SAGE Publications, 2011).

35. P. D. Lamb, *et al.*, How quantitative is metabarcoding: A meta-analytical approach. *Mol Ecol* **28**, 420–430 (2019).

36. J. Schenk, S. Geisen, N. Kleinboelting, W. Traunspurger, Metabarcoding data allow for reliable biomass estimates in the most abundant animals on earth. *MBMG* **3**, e46704 (2019).

37. M. S. Clark, Molecular mechanisms of biomineralization in marine invertebrates. *J Exp Biol* **223**, jeb206961 (2020).

SI Figures:

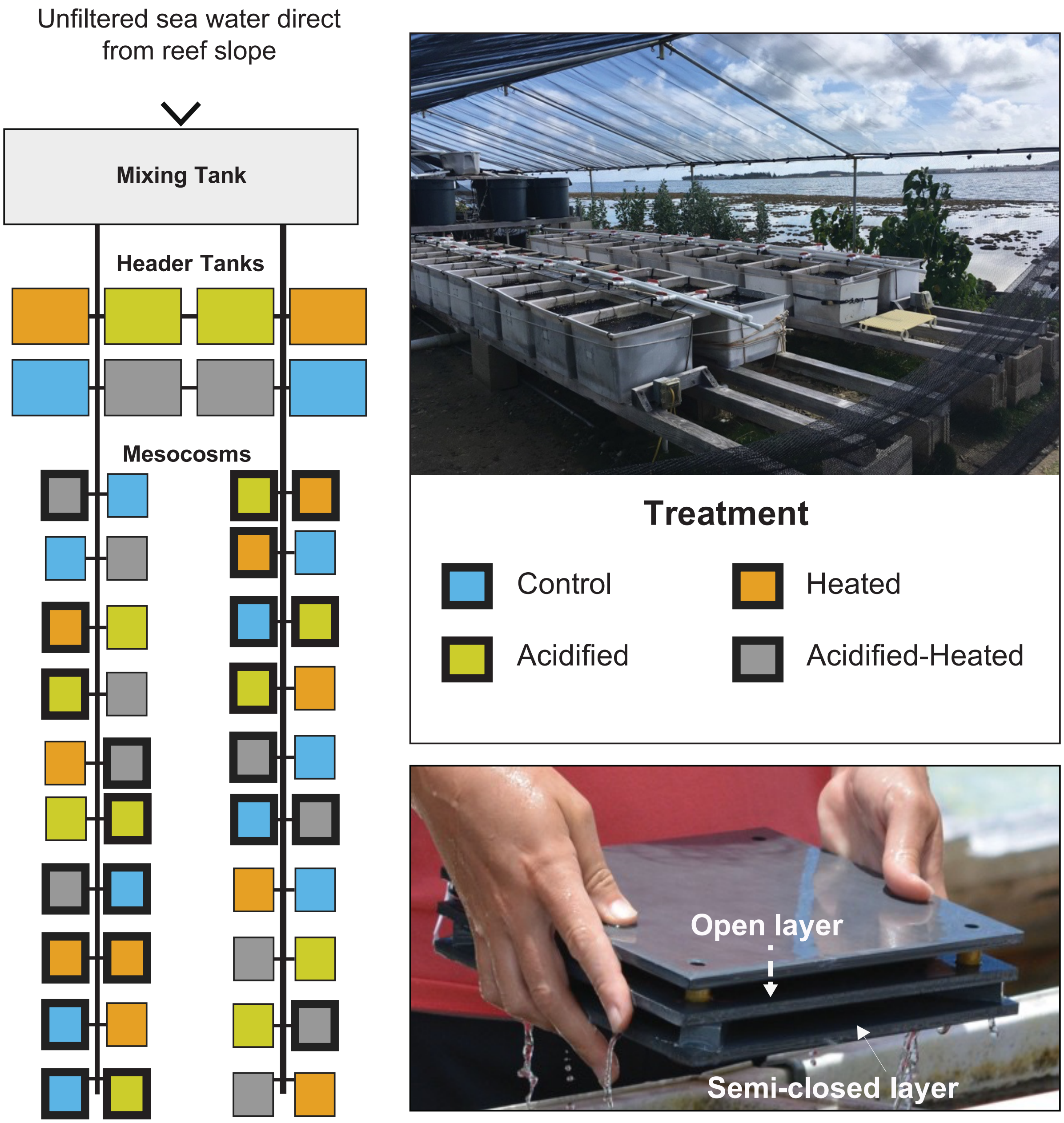

Figure S1: Schematic and image of the mesocosm system setup at the Hawaii Institute of Marine Biology. Mesocosms with ARMS units have a bolded outline. The structure of the modified ARMS unit is represented in the lower right-hand corner.

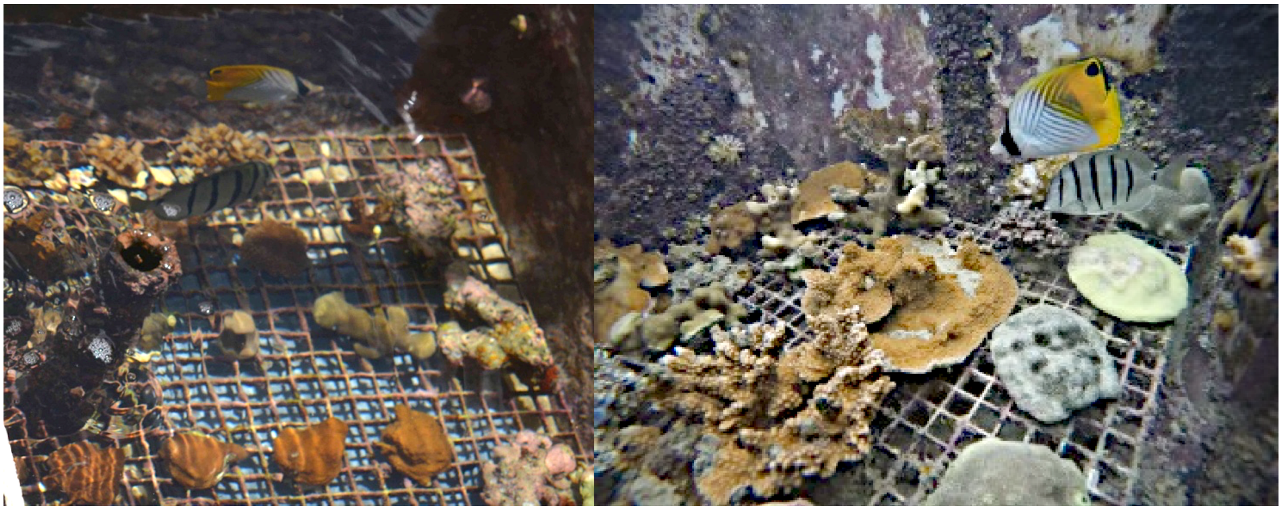

Figure S2: Representative photo of a mesocosm on the first day of the ARMS deployments (left) and after two years (right). The 2-tiered ARMS unit (see Figure S1) was placed below the grate to simulate the cryptobenthos.

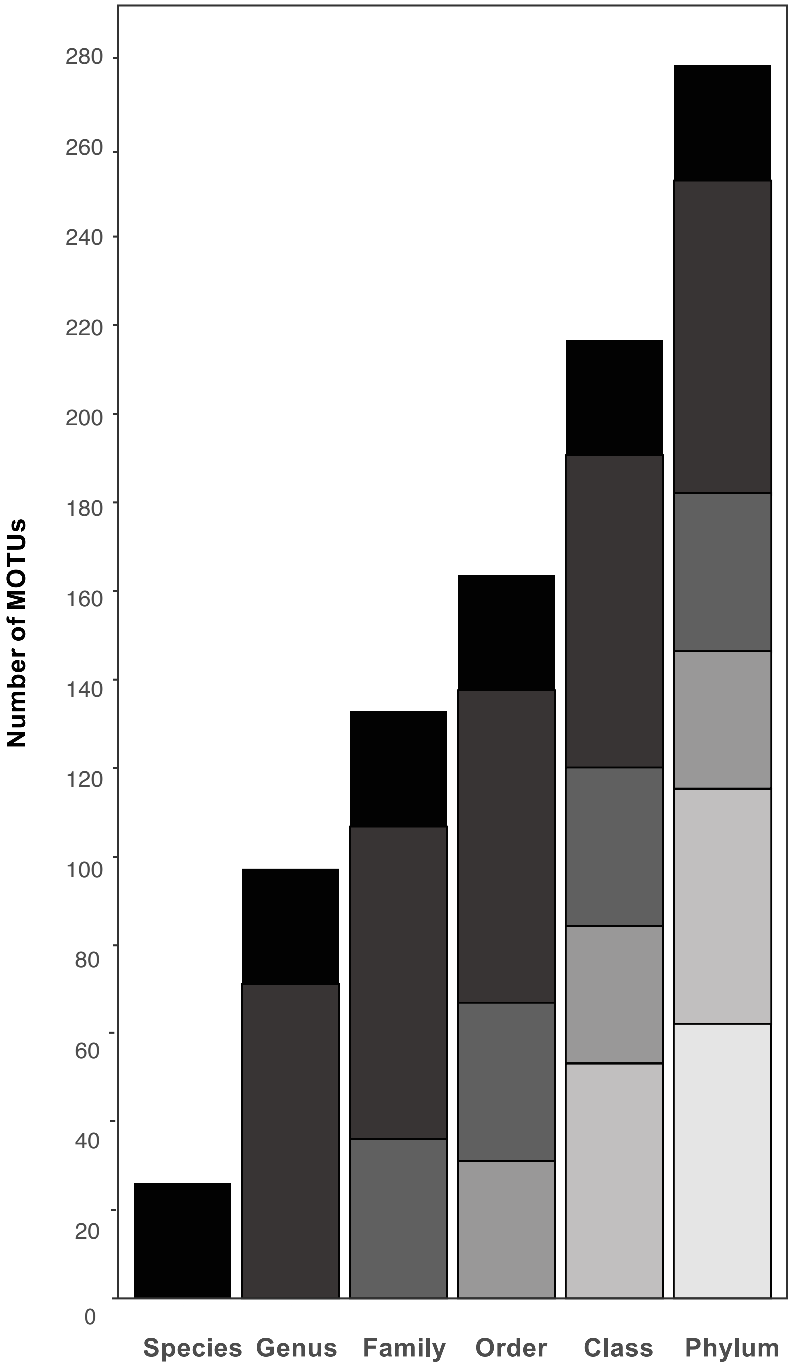

Figure S3. MOTU annotations across classification levels. Each taxon level has a representative shade of gray and is added to the next level of classification to represent the number of MOTUs identified among the 6 main classification levels. There was a total of 275 metazoan and macroalgae MOTUs contributing to this study.

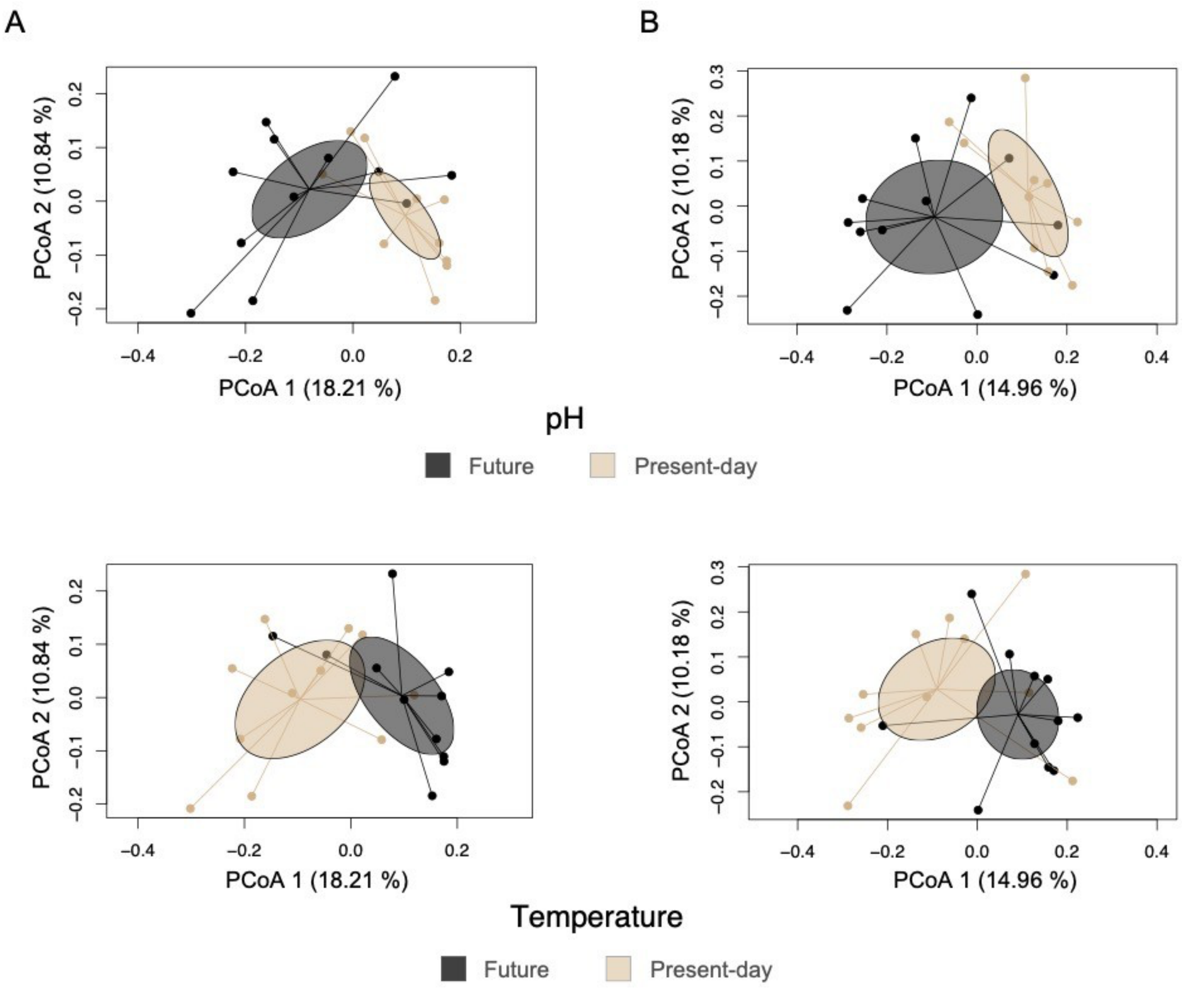

Figure S4. ARMS unit communities visualized through non-metric multi-dimensional scaling (NMDS) estimated by pH (upper panels) and temperature (lower panels), based on (A) community composition (presence/absence—Jaccard dissimilarity index) and (B) community structure (relative abundance—Bray-Curtis dissimilarity index). Ellipses are colored by future and present-day conditions as shown in the legend and based on the 95% confidence limit of the standard error around the means (SEM) for each group.

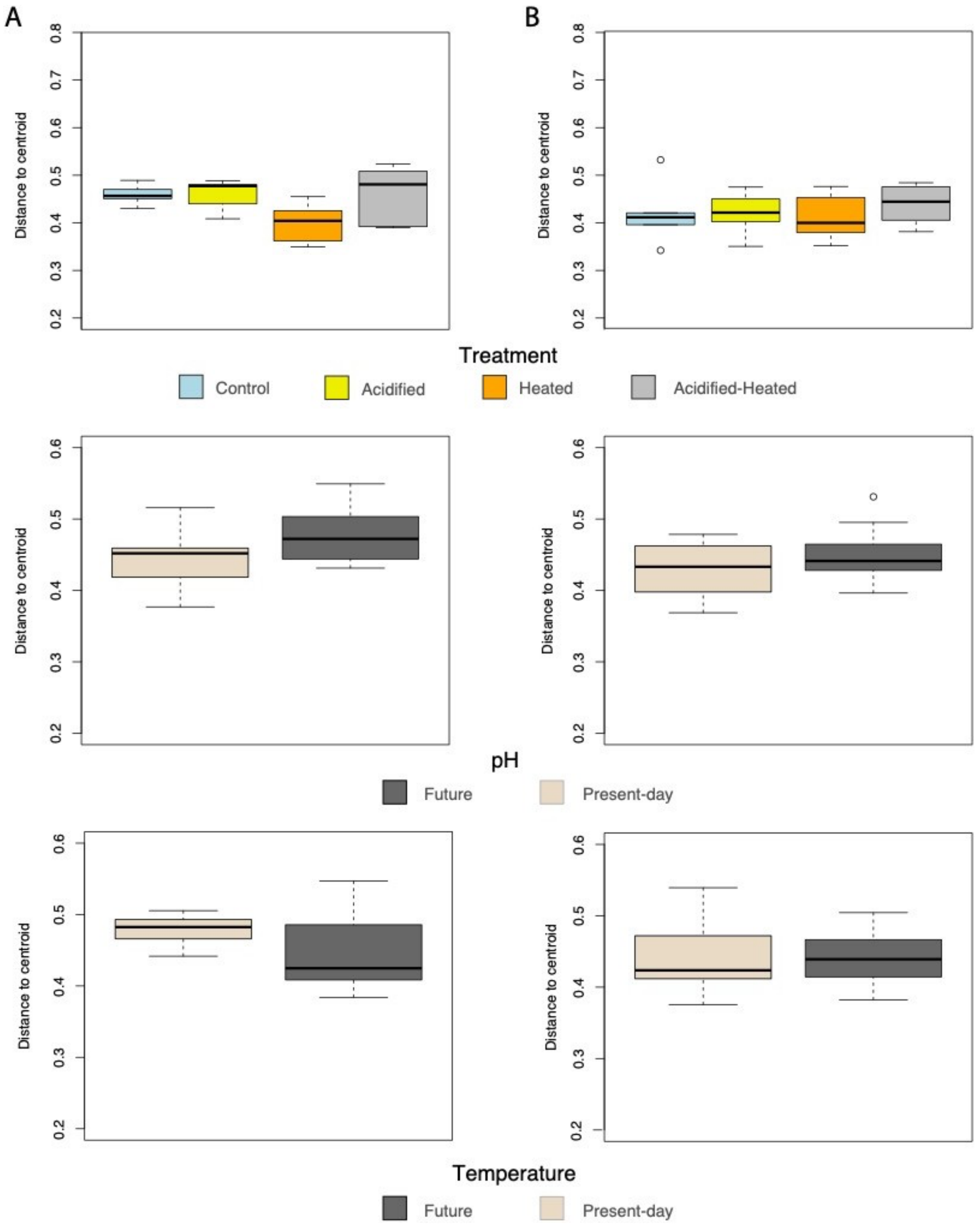

Figure S5: Boxplots of PERMDISP results examining the variation in group dispersion. estimated as (A) treatment, pH, and temperature based on the Jaccard dissimilarity index and (B) treatment, pH, and temperature based on the Bray-Curtis dissimilarity index. Box-plots show the median as center line, box limits are upper and lower quartiles, whiskers are 1.5x interquartile range, and open circles as outliers. See Table S6.

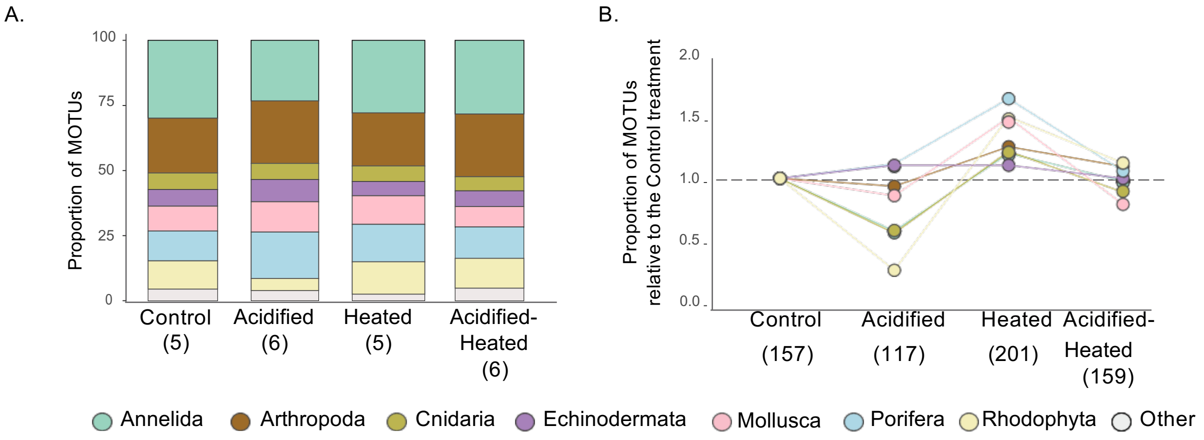

Figure S6: Variation in the top 7 phyla among treatments based on MOTUs. (A) Proportion of MOTUs by phyla within treatments. Number of sampling unit replicates in parentheses. (B) Proportion of MOTUs by phyla among treatments relative to the present-day (Control) condition. To account for sampling effects on richness, 5 randomly selected ARMS units per treatment were selected. Overall treatment richness is indicated within parentheses.

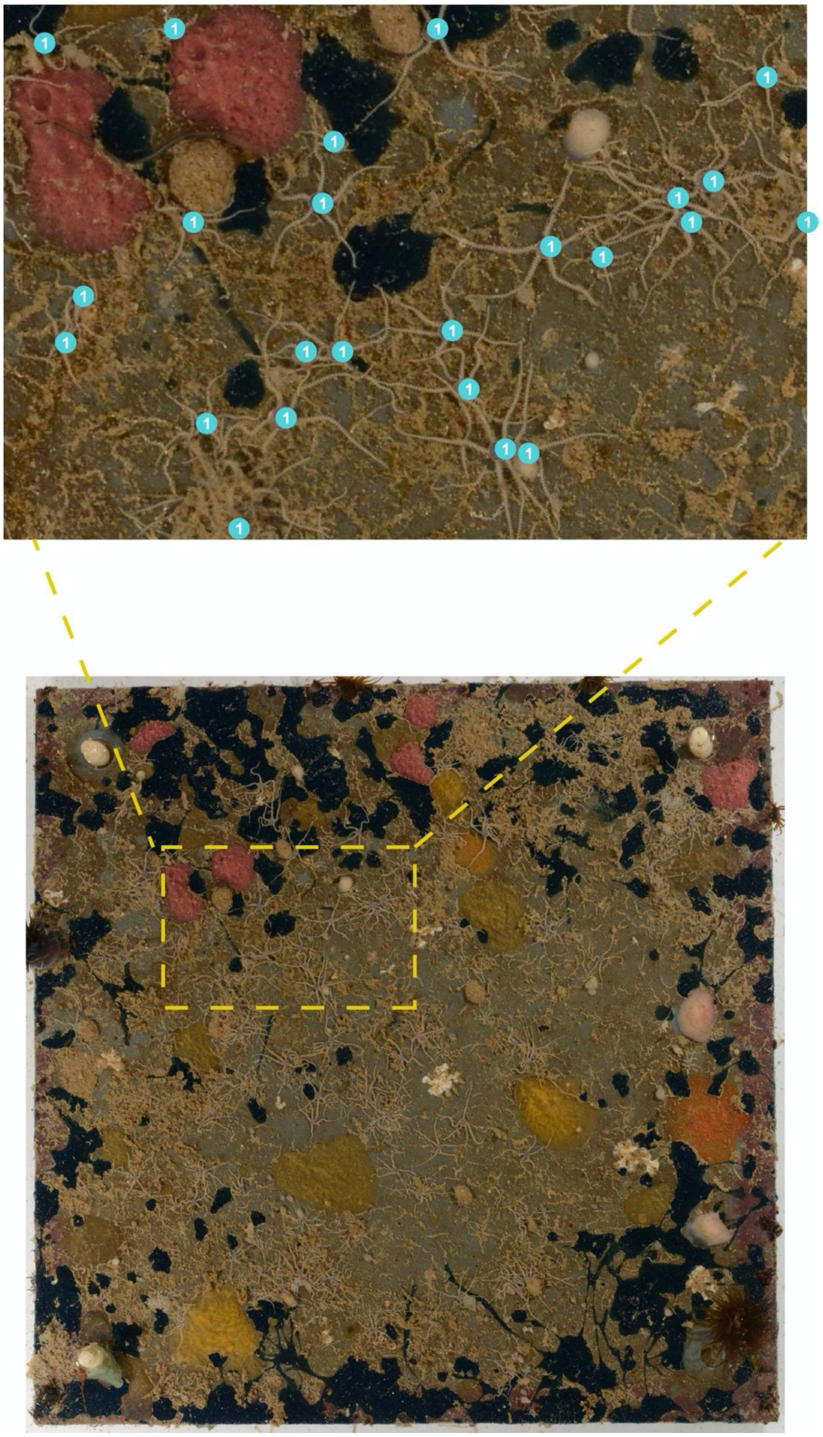

Figure S7: A plate from an ARMS that soaked in acidified conditions. Brittle stars are abundant across the entire plate. The light blue circles within the inset of a small section of the plate are placed on top of the brittle star's central disc to demonstrate their density.

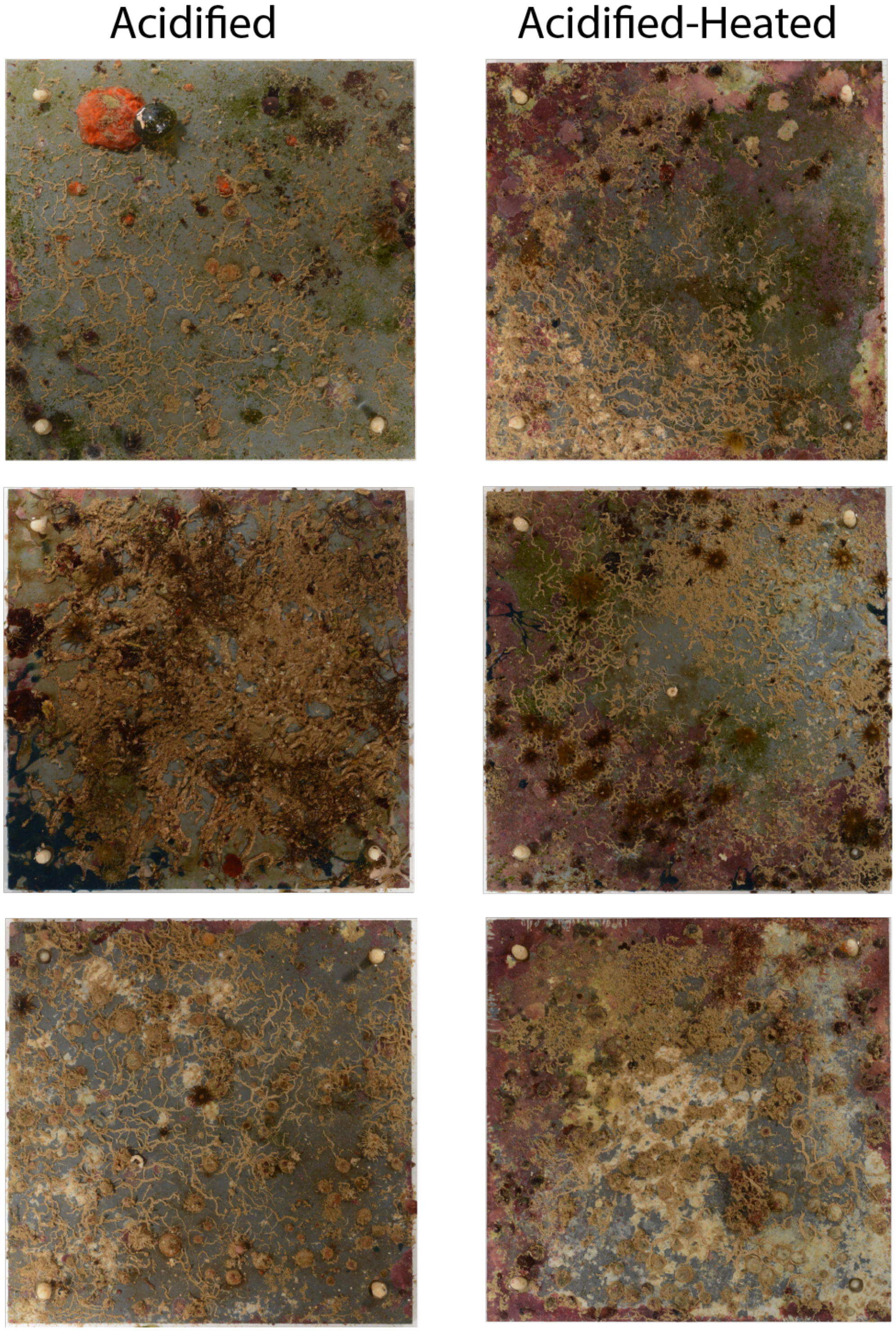

Figure S8: Representative top plates of ARMS units within the Acidified and Acidified-Heated treatments to represent the differences in red algae (Rhodophyta) among these treatments. The pink, mostly around the edges of the ARMS plates, is representative of red algae. The greater presence of red algae in the Acidified-Heated treatments compared to near absence of red algae in the Acidified treatments suggests a compensatory effect of warming.

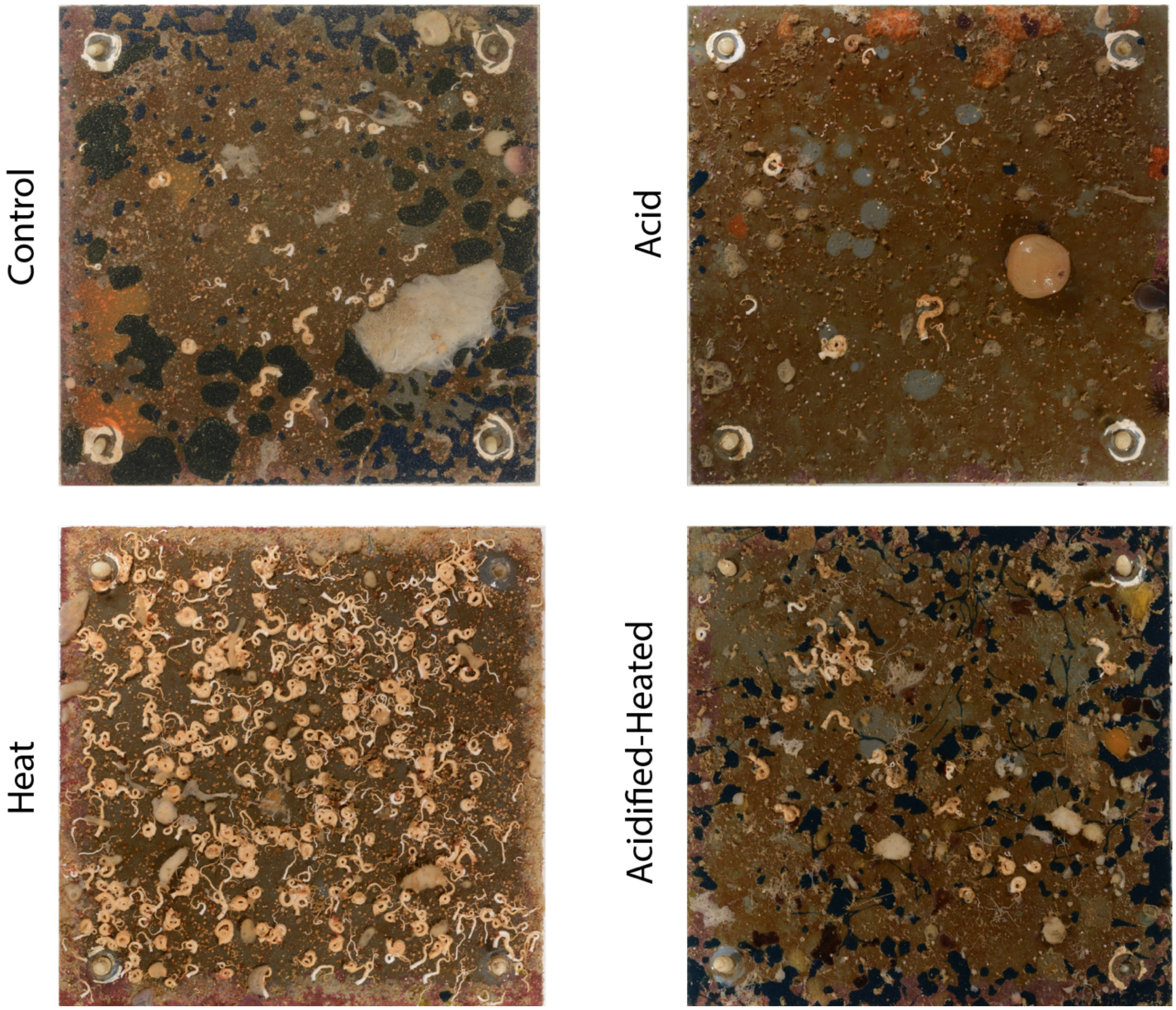

Figure S9: The bottom of a representative top plate of an ARMS unit from each treatment to demonstrate the variation of vermetid marine snails. Temperature had a positive effect on the read abundance of these marine snails.

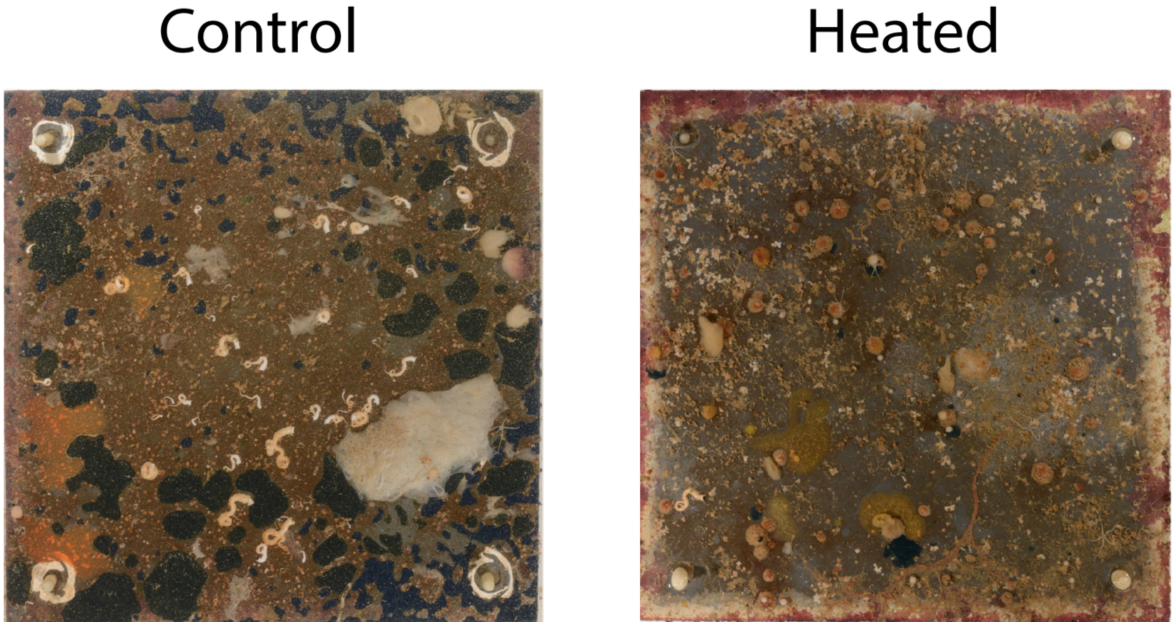

Figure S10: An ARMS plate example of sponges from the order Suberita (dark blue) from the Control and Heated treatments. Members of the Suberitidae family did not favor elevated temperature.

SI Tables:

Table S1. Sequence details associated with each ARMS unit.

Treatment	ARMS Unit	Mesocosm Name	Raw numbers of sequences	Post Filtration	Metazoa & Macroalgae	Classified to Phylum	Post Rarefied
Control							
	1	Control10	99151	98102	70588	64252	16135
	2	Control17	79252	78290	54390	43857	16137
	3	Control23	41939	41552	34504	20539	16133
	4	Control26	133359	131426	87586	69356	16133
	5	Control9	188537	186078	121869	84384	16131
Acidified	6	Acid1	147690	146290	102861	90211	16133
	7	Acid15	58971	58450	47430	38223	16133
	8	Acid25	23080	22810	16867	16132	16132
	9	Acid36	136750	135074	96455	61968	16131
	10	Acid39	87137	86311	74351	51037	16135
	11	Acid7	76079	75351	59197	54573	16132
Heated	12	Heat18	227257	222986	139375	114585	16131
	13	Heat22	108989	107753	94682	87232	16132
	14	Heat3	329916	325855	237876	133258	16135
	15	Heat31	489606	483185	429862	348066	16132
	16	Heat8	217388	214452	197158	153143	16133
Acidified-Heated	17	AcidHeat16	86486	85588	39209	34850	16130
	18	AcidHeat20	69353	68626	48010	43121	16137
	19	AcidHeat21	35957	35738	30917	26358	16127
	20	AcidHeat24	107760	106702	78706	58257	16132
	21	AcidHeat33	71096	70374	66717	35147	16135
	22	AcidHeat4	176210	174243	140163	128079	16132
	Total Sequences:		2991963	2955236	2268773	1756628	354921

Table S2. MOTU details associated with each ARMS unit.

Treatment	ARMS Unit	Mesocosm Name	Raw number of MOTUs	Post Filtration	Metazoa & Macroalgae	Classified to Phylum	Total Richness
Control	1	Control10	532	161	86	53	53
	2	Control17	613	208	119	81	81
	3	Control23	417	154	103	65	65
	4	Control26	800	217	120	82	82
	5	Control9	790	183	106	63	63
Acidified	6	Acid1	590	138	74	47	47
	7	Acid15	421	130	72	49	49
	8	Acid25	372	161	89	59	59
	9	Acid36	629	144	85	56	56
	10	Acid39	489	134	80	54	54
	11	Acid7	453	129	67	43	43
Heated	12	Heat18	1133	260	159	111	111
	13	Heat22	637	185	123	90	90
	14	Heat3	1084	226	157	97	97
	15	Heat31	1164	216	146	101	101
	16	Heat8	884	223	134	93	93
Acidified-Heated	17	AcidHeat16	501	159	77	50	50
	18	AcidHeat20	493	199	109	78	78
	19	AcidHeat21	307	167	109	81	81
	20	AcidHeat24	541	179	115	80	80
	21	AcidHeat33	480	143	99	63	63
	22	AcidHeat4	701	162	94	57	57
		Total MOTUs:	2602	859	443	275	275

Table S3. Breakdown of sequence reads and MOTUs associated with taxonomic groups.

		MOTU	Sequences
Total		2602	2,991,963
Post 0.01% MOTU Filtration		853	2,955,236
Higher Taxa Group	Bacteria	3	4759
	Metazoa	405	2,201,434
	MicoAlgae	62	21,224
	Other_Eukaryota	235	367,023
	Other_Opisthokonta	1	108
	Macroalgae	38	67,339
	Unclassified	97	270,527
Metazoa & Macroalgae	Unclassified	164	512,145
	Classified	279	1,756,628
Classified & Subsampled	Total	275	354,921
Phyla	Annelida	70	104,046
	Arthropoda	66	34,037
	Bryozoa	2	78
	Tunicata	2	636
	Cnidaria	19	16,246
	Echinodermata	11	60,665
	Gastrotricha	2	9
	Mollusca	23	64,572
	Nemertea	1	18
	Ochrophyta	5	74
	Platyhelminthes	1	209
	Porifera	41	61,120
	Rhodophyta	32	16,207

Table S4. Approximately 10% of all MOTUs identified to a species level based on ≥98% sequence similarity for all phyla minus MOTUs within the phylum Porifera which is based on 100% sequence similarity. These MOTUs do not necessarily represent the most abundant taxa from the mesocosm but rather could be identified due to available barcodes. Only 4% of MOTUs have a taxonomic species name likely because they are cosmopolitan species that have been previously studied and barcoded.

Phylum	Class	Order	Family	Genus	Species
Annelida	Polychaeta	Eunicida	Eunicidae	Nematonereis	unicornis
Annelida	Polychaeta	Terebellida	Cirratulidae	Timarete	punctata
Arthropoda	Malacostraca	Amphipoda	Gammaridae		Gammaridae sp. KML 32
Arthropoda	Malacostraca	Amphipoda	Paracalliopiidae	Yhi	yindi
Cnidaria	Anthozoa	Actiniaria	Aiptasiidae	Aiptasia	pulchella
Cnidaria	Anthozoa	Actiniaria	Sagartiidae	Sagartiogeton	laceratus
Cnidaria	Hydrozoa	Leptothecata	Campanulariidae	Clytia	simplex
Echinodermata	Ophiuroidea	Ophiurida	Ophiactidae	Ophiactis	savignyi
Echinodermata	Ophiuroidea	Ophiurida	Amphiuridae	Amphipholis	squamata
Mollusca	Gastropoda	Littorinimorpha	Hipponicidae	Antisabia	imbricata
Mollusca	Gastropoda	Littorinimorpha	Vermetidae	Dendropoma	meroclista
Mollusca	Gastropoda	Littorinimorpha	Vermetidae	Dendropoma	rhyssoconchum
Mollusca	Gastropoda	Littorinimorpha	Vermetidae	Petaloconchus	keenae
Mollusca	Gastropoda	Trochida	Trochidae	Trochus	intextus
Porifera	Demospongiae	Haplosclerida	Chalinidae	Halicona	Halicona sp.2 JV-2020
Porifera	Demospongiae	Haplosclerida	Niphatidae		Niphatidae sp.8 PRT-2020
Porifera	Demospongiae	Poecilosclerida	Tedaniidae	Tedania	klausi
Porifera	Demospongiae	Suberitida	Halichondriidae	Hymeniacidon	Hymeniacidon sp.1 JV-2020
Porifera	Demospongiae	Suberitida	Halichondriidae	Halichondria	Halichondria sp.1 JV-2020
Porifera	Demospongiae	Suberitida	Suberitidae		Suberitidae sp.1 JV-2020
Porifera	Demospongiae	Suberitida	Suberitidae	Terpios	Terpios sp.1 JV-2020
Porifera	Demospongiae	Tethyida	Tethyidae	Tethya	Tethya sp.2 JV-2020
Porifera	Demospongiae	Tethyida	Tethyidae	Tethya	Tethya sp.3 JV-2020
Porifera	Homoscleromorpha	Homosclerophorida	Plakinidae	Plakina	Plakina sp.1 JV-2020
Porifera	Homoscleromorpha	Homosclerophorida	Plakinidae	Plakortis	Plakortis sp.1 JV-2020
Porifera	Homoscleromorpha	Homosclerophorida	Oscarellidae	Oscarella	Oscarella sp.3 JV-2020

Table S5. Richness summary statistics by treatment.

Treatment	ARMS units	Mean	sd
Control	5	68.8	12.5
Acidified	6	51.3	6.02
Heated	5	98.4	8.17
Acidified-Heated	6	68.2	13.3

Table S6. A two-way ANOVA on the effects of elevated temperature (T), acidification (A) and their interaction (T×A) nested within the header tank (Header) on observed richness.

Factors	Df	SS	MS	F	P
T	1	2818	2818	27.27	**< 0.001**
A	1	3102	3102	30.02	**< 0.001**
T x A	1	222	222	2.15	0.165
Header (T x A)	4	505.2	126.3	1.22	0.345
Residuals	14	1447	103.4		

Df = degrees of freedom, SS = sum of squares, MS = mean squares, F = the F-value, and P = the probability. Bolded p-values indicates significance at $p < 0.05$.

Table S7. Pairwise post-hoc lsmeans method comparing a family of 4 estimates (presence/absence of elevated temperature and acidification conditions) translated into treatment names averaged over header tank with observed richness.

Treatments	estimate	SE	df	t.ratio	p-adj
Acidified & Heated	-46.58	6.23	14	-7.482	**<0.001**
Acidified & Acidified-Heated	-16.83	5.87	14	-2.868	0.054
Acidified-Heated & Heated	29.75	6.23	14	4.779	**0.002**
Control & Acidified	18.25	6.23	14	2.931	**0.047**
Control & Heated	-28.33	6.56	14	-4.318	**0.004**
Control & Acidified-Heated	1.45	6.23	14	0.228	0.996

Diff = the difference in the observed means, lwr = the lower end point, upr = upper end point, bold p-adj indicates significance at $p < 0.05$ and gray p-adj indicates values at $p < 0.1$.

Table S8. Pairwise Permutational Multivariate Analysis of Variance (PERMANOVA) of the community response between treatments.

Treatment	Metric	R2	p-adj
Acidified & Acidified-Heated	Bray-Curtis	0.17	**0.01**
	Jaccard	0.15	**<0.01**
Acidified & Heated	Bray-Curtis	0.24	**0.01**
	Jaccard	0.25	**<0.01**
Acidified-Heated & Heated	Bray-Curtis	0.15	**0.01**
	Jaccard	0.16	**<0.01**
Control & Acidified	Bray-Curtis	0.19	**0.01**
	Jaccard	0.16	**<0.01**
Control & Heated	Bray-Curtis	0.16	**0.05**
	Jaccard	0.17	**<0.01**
Control & Acidified-Heated	Bray-Curtis	0.14	**0.05**
	Jaccard	0.09	0.62

The permutational probability based on 9999 permutations with a false discovery rate adjusted to the p-value. Bold p-adj indicate a significance at $p < 0.05$.

Table S9. Permutational Analysis of Multivariate Dispersion (PERMDISP) on treatments and elevated temperature and increased pH conditions.

Metric	Factors	Df	SS	Pseudo-F	P
Bray-Curtis	Treatment	3	0.002	0.28	0.84
	Temperature	1	<0.001	0.07	0.80
	pH	1	0.002	1.69	0.22
Jaccard	Treatment	3	0.015	2.83	0.09
	Temperature	1	0.003	1.74	0.20
	pH	1	0.007	3.94	0.06

Degrees of freedom (DF), sum of squares (SS), the pseudo F-value (Pseudo-F), and the permutational probability (P) based on 9999 permutations. Significance is based on $p < 0.05$. Gray values represent $p < 0.1$

Table S10. Pairwise Permutational Analysis of Multivariate Dispersion (PERMDISP) on treatments.

Treatment	Metric	p-adj
Acidified & Acidified-Heated	Bray-Curtis	0.59
	Jaccard	0.99
Acidified & Heated	Bray-Curtis	0.78
	Jaccard	**0.02**
Acidified-Heated & Heated	Bray-Curtis	0.36
	Jaccard	0.08
Control & Acidified	Bray-Curtis	0.99
	Jaccard	0.86
Control & Heated	Bray-Curtis	0.83
	Jaccard	**0.02**
Control & Acidified-Heated	Bray-Curtis	0.59
	Jaccard	0.90

The permutational probability based on 9999 permutations with a false discovery rate adjusted to the p-value. Bold p-adj indicate significance at $p < 0.05$.

Table S11. Proportion of phlya reads within each treatment relative to the Control condition.

Phylum	Total Reads per Phylum	Treatment	Reads per Treatment	Relative Abundance of Reads	Proportion of Reads Relative to Control
Annelida	91261	Control	26166	28.67	1.00
		Acidified	26758	29.32	1.02
		Heated	18412	20.18	0.70
		Acidified-Heated	19925	21.83	0.76
Arthropoda	31372	Control	3549	11.31	1.00
		Acidified	11427	36.42	3.22
		Heated	6738	21.48	1.90
		Acidified-Heated	9658	30.79	2.72
Cnidaria	13902	Control	4011	28.85	1.00
		Acidified	3391	24.39	0.85
		Heated	3597	25.87	0.90
		Acidified-Heated	2903	20.88	0.72
Echinodermata	54431	Control	9822	18.04	1.00
		Acidified	20736	38.10	2.11
		Heated	5894	10.83	0.60
		Acidified-Heated	17979	33.03	1.83
Mollusca	57399	Control	13120	22.86	1.00
		Acidified	5526	9.63	0.42
		Heated	25929	45.17	1.98
		Acidified-Heated	12824	22.34	0.98
Porifera	58051	Control	19886	34.26	1.00
		Acidified	12582	21.67	0.63
		Heated	16508	28.44	0.83
		Acidified-Heated	9075	15.63	0.46
Rhodophyta	15314	Control	3825	24.98	1.00
		Acidified	155	1.01	0.04
		Heated	3542	23.13	0.93
		Acidified-Heated	7792	50.88	2.04

Table S12. Proportion of MOTUs within each treatment relative to the Control condition.

Phylum	Total MOTUs per Phylum	Treatment	MOTUs per Treatment	Relative Abundance of MOTUs	Proportion of MOTUs Relative to Control
Annelida	73	Control	47	64.38	1.00
		Acidified	28	38.36	0.60
		Heated	56	76.71	1.19
		Acidified-Heated	46	63.01	0.98
Arthopoda	66	Control	33	50.00	1.00
		Acidified	31	46.97	0.94
		Heated	41	62.12	1.24
		Acidified-Heated	38	57.58	1.15
Cnidaria	19	Control	10	52.63	1.00
		Acidified	6	31.58	0.60
		Heated	12	63.16	1.20
		Acidified-Heated	8	42.11	0.80
Echinodermata	11	Control	10	90.91	1.00
		Acidified	11	100.00	1.10
		Heated	11	100.00	1.10
		Acidified-Heated	10	90.91	1.00
Mollusca	24	Control	15	62.50	1.00
		Acidified	13	54.17	0.87
		Heated	22	91.67	1.47
		Acidified-Heated	13	54.17	0.87
Porifera	41	Control	18	43.90	1.00
		Acidified	20	48.78	1.11
		Heated	29	70.73	1.61
		Acidified-Heated	19	46.34	1.06
Rhodophyta	32	Control	17	53.13	1.00
		Acidified	5	15.63	0.29
		Heated	25	78.13	1.47
		Acidified-Heated	19	59.38	1.12

Table S13. A two-way permutational ANOVA on the effects of elevated temperature (T), acidification (A) and their interaction (T x A) on the top 7 phyla.

Phylum	Factors	Df	SS	MS	F	Pr(>F)
Annelida	T	1	12.75	12.75	1.68	0.20
	A	1	0.27	0.27	0.04	0.87
	T x A	1	0.05	0.05	0.01	0.94
	Residuals	16	121.01	7.56		
Arthropoda	T	1	1.03	1.03	0.07	0.79
	A	1	59.11	59.141	4.34	**0.05**
	T x A	1	12.68	12.68	0.93	0.37
	Residuals	16	217.99	13.62		
Cnidaria	T	1	2.02	2.02	0.05	0.84
	A	1	4.47	4.47	0.11	0.76
	T x A	1	0.02	0.02	0.00	0.98
	Residuals	16	655.69	40.98		
Echinodermata	T	1	7.65	7.65	0.70	0.41
	A	1	89.35	89.35	8.22	**0.01**
	T x A	1	0.22	0.22	0.02	0.87
	Residuals	16	176.86	10.87		
Mollusca	T	1	64.99	64.99	3.41	0.11
	A	1	61.42	61.41	3.22	0.11
	T x A	1	4.60	4.60	0.24	0.66
	Residuals	16	304.99	19.06		
Porifera	T	1	7.04	7.04	0.24	0.70
	A	1	32.35	32.35	1.08	0.34
	T x A	1	0	0.00	0.00	0.99
	Residuals	16	477.63	29.85		
Rhodophyta	T	1	115.24	115.24	3.34	0.08
	A	1	0.74	0.74	0.02	0.89
	T x A	1	134.31	134.21	3.89	0.06
	Residuals	16	552.32	34.52		

Permutations = 999, Df = degrees of freedom, SS = sum of squares, MS = mean squares, F = the F-value, and P = the probability. Bolded p-values indicates significance at $p < 0.05$ and gray values represent $p < 0.1$

Table S14. A two-way permutational ANOVA on the effects of elevated temperature (T), acidification (A) and their interaction (T x A) on the top 8 families.

Phylum	Family	Factors	Df	SS	MS	F	Pr(>F)
Annelida	Amphinomidae	T	1	10.76	10.76	0.23	0.61
		A	1	51.18	51.17	1.07	0.34
		T x A	1	18.39	18.39	0.38	0.56
		Residuals	16	760.73	47.55		
	Cirratulidae	T	1	2.79	2.79	0.04	0.89
		A	1	364.99	364.99	4.79	**0.02**
		T x A	1	20.83	20.82	0.27	0.60
		Residuals	16	1218.49	76.16		
Arthropoda	Gammaridae	T	1	7.614	7.61	0.56	0.49
		A	1	154.94	154.94	11.43	**<0.01**
		T x A	1	2.77	2.77	0.20	0.68
		Residuals	16	216.92	13.56		
Echinodermata	Amphiuridae	T	1	11.34	11.34	0.68	0.43
		A	1	51.48	51.48	3.10	0.09
		T x A	1	2.05	2.05	0.12	0.71
		Residuals	16	265.45	16.59		
	Ophiactidae	T	1	46.82	46.82	1.99	0.18
		A	1	120.27	120.27	5.11	**0.04**
		T x A	1	3.02	3.02	0.13	0.73
		Residuals	16	376.63	23.54		
Mollusca	Hipponiccidae	T	1	7.79	7.79	0.24	0.63
		A	1	197.39	197.39	6.12	**0.02**
		T x A	1	8.12	8.12	0.25	0.61
		Residuals	16	516.34	32.27		
	Vermetidae	T	1	137.13	137.13	4.88	**0.05**
		A	1	40.32	40.32	1.44	0.23
		T x A	1	10.05	10.05	0.36	0.56
		Residuals	16	449.18	28.07		
Porifera	Suberitidae	T	1	114.31	114.31	3.75	0.06
		A	1	7.84	7.84	0.26	0.60
		T x A	1	14.46	14.46	0.47	0.50
		Residuals	16	487.39	30.46		

Permutations = 999, Df = degrees of freedom, SS = sum of squares, MS = mean squares, F = the F-value, and P = the probability. Bolded p-values indicates significance at $p < 0.05$ and gray values represent $p < 0.1$
